# The economically optimal mix and timing of coastal adaptation in Europe to 2150

**DOI:** 10.1038/s41467-026-74042-8

**Published:** 2026-07-15

**Authors:** Vanessa Völz, Jochen Hinkel, Daniel Lincke, Lars E. Honsel, Robert J. Nicholls, Rémi Thiéblemont, Gonéri Le Cozannet, Paul Sayers

**Affiliations:** 1 Thaer-Institute of Agricultural and Horticultural Sciences, Humboldt-Universität zu Berlin, Berlin, Germany; 2 Global Climate Forum, Berlin, Germany; 3 Tyndall Centre for Climate Change Research, University of East Anglia (UEA), Norwich, UK; 4 BRGM - French Geological Survey, Orléans, France; 5 Sayers and Partners, Watlington, UK

**Keywords:** Natural hazards, Climate-change adaptation, Environmental economics, Climate-change impacts

## Abstract

Coastal adaptation to sea level rise involves combining and sequencing adaptation options into adaptation pathways that include adaptation tipping points, which are moments requiring a switch between options, often due to economic limits. While local case studies show costs depend on the economically optimal timing of actions and adaptation tipping points, this remains unexplored on global and continental scales. We present economically optimal adaptation pathways for Europe’s coastal floodplains through 2150. For 95% of the coastline requiring adaptation, the optimal timing for initial actions is immediate due to current economic under-adaptation, a condition in which the costs of additional adaptation are outweighed by the reduction in expected flood damages. By 2150, retreat is economically optimal for 22% of the coastline, incurring lower total costs than protection, which is optimal for 9%. Adaptation tipping points are primarily driven by sea level rise and necessitate a switch from accommodation to either protection or retreat, or from retreat to inland protection combined with retreat in the foreland. Their timing depends on climate change, occurring 32 years earlier under higher climate change scenarios compared to lower ones.

## Abstract (web summary)

Coastal adaptation to sea level rise requires timely action, but global economic evidence is limited. This study shows that one third of Europe’s coastline benefits from immediate adaptation through retreat, protection and accommodation.

## Introduction

Within the coming decades, massive investments in coastal adaptation need to be made in Europe because accelerating sea level rise (SLR) exacerbates coastal flood risks, posing serious dangers to about 50 million people residing in Europe’s low-lying coastal areas^1,2^. Between 1971 and 2018, global sea levels rose by 11 cm, with an accelerating trend especially from 2006 onwards^3^. Future projections indicate that 50 cm of SLR will be reached during the second half of this century irrespective of how successful greenhouse gas emissions (GHG) are reduced^4^. Exceeding higher values of SLR is not a question of if but when^5^, and if substantive GHG reduction fails, Europe and the rest of the world could face a SLR of 1 meter or more by 2100^3^.

Against this backdrop, the latest assessments of the Intergovernmental Panel on Climate Change (IPCC) have drawn three conclusions^1,6^. First, coastal adaptation needs to consider a wide range of adaptation options, including protection (e.g., dikes, restoring wetlands, storm surge barriers), accommodation (e.g., flood-proofing or elevating buildings) and retreat (i.e., moving inland)^1,7^. There is no one-size-fits-all option, but all options have a range of advantages and disadvantages that need to be considered given the local context.

Second, adaptation can not be a single-stage action, but options must be combined and sequenced over time into adaptation pathways. Crucially, such pathways potentially entail adaptation tipping points (ATP), moments or conditions in time when it is necessary to switch from one option to another, such as, e.g., from protection to retreat^8^. Consequently, an increasing number of local case studies developed adaptation pathways highlighting when ATP occur^9^. Experiences from those local studies have been combined by IPCC authors into generic adaptation pathways illustrating ATP for different coastal archetypes^7,10,11^.

A third conclusion is that ATP occurs generally not due to biophysical or technical limits, but rather due to economic and social limits^1,7^. Coastal protection technology is mature and has been applied for decades to centuries for protecting land^12^, including in locations, such as the coastal marshlands of Germany and the Netherlands, as well as major delta cities like Tokyo and Jakarta, which have experienced several meters of SLR due to human-induced land subsidence^13^. Such technology, however, is costly and requires large and long-term public infrastructure investments^14^. Hence, the application of economic decision-making tools (e.g., cost-benefit analysis) is widespread or even legally prescribed because governments have a fiduciary duty to spend tax payers money wisely^15,16^. While this study emphasizes economic ATPs, the original concept was defined more broadly as situations where the magnitude of change prevents an option from meeting its objectives^8^. In the literature, ATPs are most often associated with risk thresholds, but they may also result from physical, ecological, technical, societal, political, or - as explored here - economic limits^9^.

These three important conclusions have yet to be incorporated into large-scale assessments, as, to our knowledge, no global or continental-scale coastal impact and adaptation assessment has considered economic ATP by incorporating the timing of adaptation actions. Most previous large-scale assessments have ignored economic considerations and implemented a predefined adaptation scenario (e.g., maintain the dike height over time, maintain a given safety standard over time)^17–24^. Some assessments have conducted a single-stage cost-benefit analysis, i.e., minimizing the discounted sum of costs and benefits over a given time horizon, but assuming that adaptation actions are implemented today and remain constant over the time horizon considered (e.g., maintain a constant protection level over time)^25–32^. These assessments thus ignore ATP and the timing of adaptation actions, despite evidence from local coastal adaptation case studies showing that adaptation costs are influenced by these factors^33–36^. Moreover, non-economic global assessments consider adaptation tipping points and highlight the need for timely adaptation from a risk perspective in the coming decades^37,38^.

We offer large-scale quantitative evidence on adaptation pathways for all of coastal Europe, thereby identifying economically optimal coastal adaptation pathways, including economic ATP, for each individual floodplain. Here, economically optimal refers to the adaptation option that minimizes the combined costs of flood damages and adaptation actions from a public economic perspective. We consider 41,327 coastal floodplains in a time horizon from 2020 to 2150, a discount rate of 3% (with sensitivity analysis for 1 and 5%), and three greenhouse gas emission scenarios: low emissions (SSP1-2.6), medium emissions (SSP2-4.5), and very high emissions (SSP5-8.5). We consider three distinct adaptation options: protection, retreat, and accommodation, as well as their combinations. Protection involves implementing hard defensive infrastructure along the coast to an appropriate height; retreat entails relocating people and assets from the floodplain; and accommodation involves flood-proofing buildings within the floodplain to withstand extreme events without damage.

Our results provide an updated and more realistic estimation of costs for coastal adaptation and residual expected flood damages in Europe. This addresses limitations of previous single-stage cost-benefit analyses, which tend to overestimate costs by assuming constant adaptation options despite rising sea levels, thereby leading to both over- and under-adaptation over time^34,39,40^. The quantitative adaptation pathways derived here identify where, when, and which coastal adaptation option is economically optimal, as well as where, when, and for which options ATP occur. Thus, they can be used to verify the qualitative expert-judgment-based work on generic adaptation pathways. While these insights into the economically optimal sequencing and timing of adaptation options provide a crucial source of information for European and global-level policy decisions^41^, and offer a first-order assessment of what may be locally economically optimal, we note that economic considerations can and should not be the only type of information used in coastal decisions.

## Results

### Economically optimal adaptation pathways

**Fig. 1:**
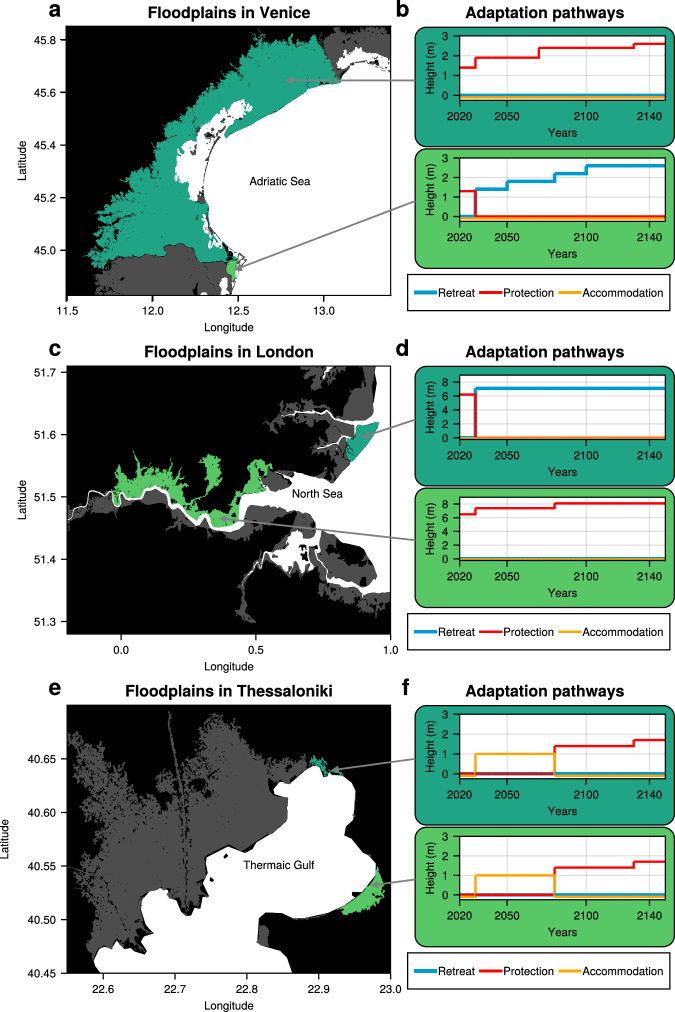
Economically optimal adaptation pathways for selected floodplains in Venice, London and Thessaloniki under SSP2-4.5. **a**,**c**,**e** The panels on the left show the location of the two floodplains within (**a**) Venice, (**c**) London and (**e**) Thessaloniki. **b**,**d**,**f** The panels on the right show the corresponding economically optimal adaptation pathway, in terms of the heights of protection, retreat and accommodation options over time. Here, the height of retreat refers to the height above sea level below which assets and people are removed. Eurostat (GISCO), 2024. Licensed under CC BY 4.0: https://creativecommons.org/licenses/by/4.0/ⓒEuroGeographics for administrative boundaries in the basemaps.

To illustrate the general nature of our approach, we first present results for three major European cities under SSP2-4.5, representing different geographic and socio-economic characteristics in terms of extreme sea levels, population density, and current protection levels. The cities are: London, a mega-city with very high coastal flood protection; Venice, a large city with medium protection levels and UNESCO World Heritage status; and Thessaloniki, a large city without large coastal flood protection today. In our model, each city and its surrounding regions include multiple floodplains, spanning densely populated urban areas as well as rural zones with low asset values. We focus on two representative floodplains per city (Fig. 1).

In Venice and London, the economically optimal adaptation pathways involve protection for the largest and most central floodplains, with incremental increases in protection heights over time. London requires higher protection heights than Venice due to its larger concentration of assets and higher extreme sea levels. London’s city center is currently protected against a 1-in-1000 year flood event (6 m above mean sea level), while Venice is protected against a 1 in 100-year flood event (1.5 m above mean sea level). In proximity to major urban centers, such as London and Venice, retreat is an economically optimal option for floodplains containing low asset values, such as those used for agriculture, nature reserves, or green space. The largest floodplains of this kind identified for retreat in our three examples are located east of London on the outer Thames Estuary marshes and south of Venice in the Po Estuary, spanning coastal lengths of 37 km and 38 km, respectively. In these areas, current protection is maintained until 2030, after which retreat is implemented (Fig. 1). London has an adaptive plan for coastal flooding over the next century^42^. While the detailed proposals cannot be compared with the results of this model, qualitatively there is good agreement with this analysis showing incremental upgrades of protection levels at a rate depending on the rate of sea-level rise, leading to comparable overall outcomes.

In contrast, two currently unprotected floodplains in Thessaloniki, covering the port in the city center and the airport to the east, experience higher relative SLR due to land subsidence. For these areas, accommodation through flood-proofing buildings up to one meter is economically optimal in 2030 and remains so until 2080. At this point, adaptation tipping points (ATP) are reached, requiring a switch from accommodation to protection, which serves as the final adaptation option until 2150 for both floodplains.

In all six adaptation pathways across the two representative floodplains per city (Fig. 1), the very first adaptation actions are implemented in 2030. These actions include upgrading protection heights in densely populated floodplains (London and Venice), switching from protection to retreat in rural floodplains (near London and Venice), and implementing accommodation in floodplains in Thessaloniki.

### When should we start adapting?

**Fig. 2:**
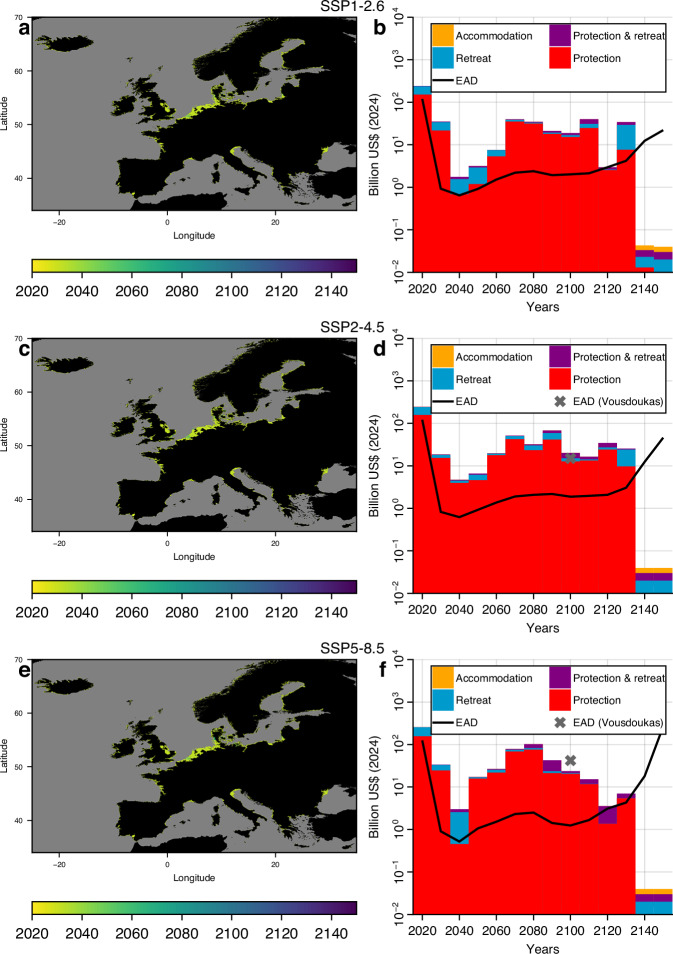
The timing of adaptation actions. **a**,**c**,**e** Maps showing the year of implementation for the very first adaptation action for each floodplain under (**a**) SSP1-2.6, (**c**) SSP2-4.5 and (**e**) SSP5-8.5. **b**,**d**,**f** Graphs showing adaptation investments for each adaptation option (stacked bar plots) and expected annual flood damage (EAD) (black line), shown undiscounted, over time and aggregated across all floodplains on a logarithmic scale, under (**b**) SSP1-2.6, (**d**) SSP2-4.5 and (**f**) SSP5-8.5. EAD (Vousdoukas) indicates the European EAD reported in Vousdoukas et al.^29^. Eurostat (GISCO), 2024. Licensed under CC BY 4.0: https://creativecommons.org/licenses/by/4.0/ⓒEuroGeographics for administrative boundaries in the basemaps.

We find that immediate action in the first time step (i.e., 2030) is required for 95% of the coastline needing adaptation across all climate change scenarios (Fig. 2), aligning with urgent calls for prompt adaptation actions from the IPCC^1^. In floodplains where coastal retreat is economically optimal, it is almost always (99% of the retreated coast length) economically advantageous to make this decision in the first time step (i.e., 2030), before asset values and retreat costs rise due to GDP growth (Fig. 2). Assuming current protection levels are 20% higher in a sensitivity analysis, 5% of the European coastline requiring adaptation could delay its first adaptation actions until 2050 or later, compared to only 2% under the baseline scenario (see section on sensitivity analysis).

Immediate adaptation actions can be attributed to the vulnerability and exposure of coastal floodplains, which are already locked into existing conditions and are currently under-adapted below the economically optimal adaptation level, because over two-thirds of immediate adaptation investments are required even in a counterfactual scenario without socioeconomic development (SED) (Figs. S2 and S3).

### Which adaptation options should we choose?

**Fig. 3:**
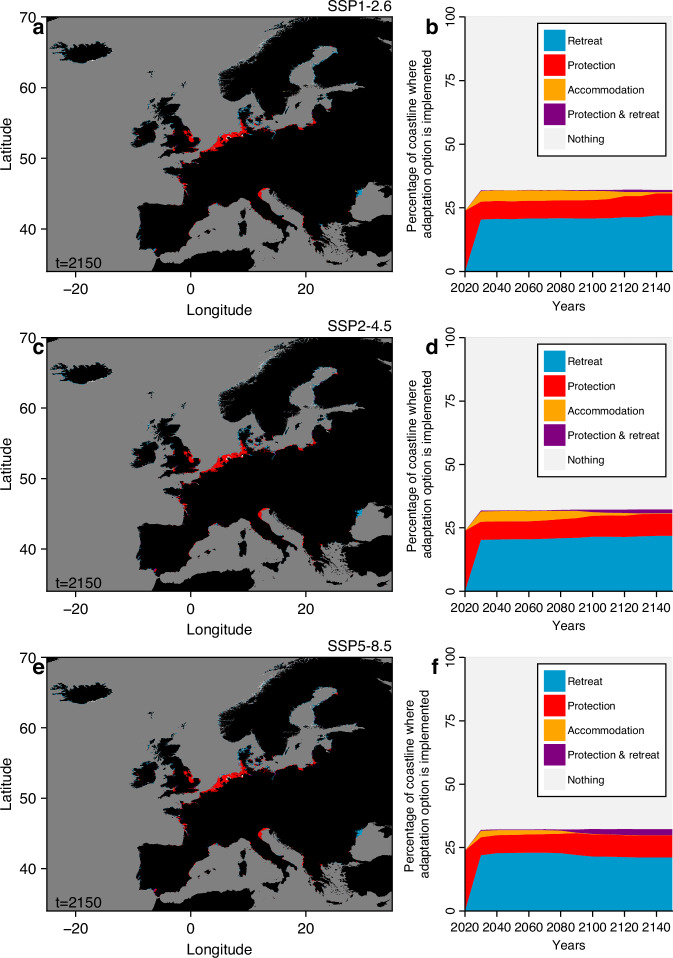
Implemented adaptation options across floodplains and over time. **a**,**c**,**e** Maps showing the final adaptation option implemented per floodplain in 2150 under **a** SSP1-2.6, **b** SSP2-4.5 and **c** SSP5-8.5. **b**,**d**,**f** Graphs showing the percentage of the entire European coastline where each adaptation option is implemented over time under **b** SSP1-2.6, **d** SSP2-4.5 and **f** SSP5-8.5. Eurostat (GISCO), 2024. Licensed under CC BY 4.0: https://creativecommons.org/licenses/by/4.0/ⓒEuroGeographics for administrative boundaries in the basemaps.

Retreat is the economically optimal response for 22% of the total length of Europe’s coastline to address rising sea levels and increasing flood risks under SSP2-4.5 in 2150 (Table 1 and Fig. 3). Protection is economically optimal for 9% of the total length of Europe’s coastline (Table 1), particularly in large, low-lying, and densely populated floodplains as found in the Netherlands, Northwest Germany, Belgium, the Venice Lagoon, and Eastern UK (Fig. 3), where protection has a long history^43^. For 68% of the total length of Europe’s coastline, no adaptation options are required, irrespective of the climate change scenario, as these steep coastal areas are not exposed to flood risks and SLR (Table 1, S1 and S2). The percentage of coastline where retreat or protection is implemented in 2150 is most influenced by varying adaptation costs and the discount rate, while different climate change scenarios have only a minor influence (see section on sensitivity analysis).

**Table 1:** The proportion of the coastline for which different adaptation options are implemented in 2150, along with the net present value of costs

Country	Coast length (km)	Protection (%)	Retreat (%)	Accommo-dation (%)	Protect and retreat (%)	Nothing (%)	Adaptation costs in 2020 (bil. US$)	Adaptation costs in 2020 (% of 2023 GDP)	Total adaptation costs (bil. US$)	Total residual flood damage (bil. US$)
Åland	3886	0.0	5.2	0.0	0.0	94.7	0.0	–	0.2	0.0
Albania	753	43.6	10.9	0.0	0.0	45.5	0.7	2.9	1.4	25.4
Belgium	319	96.5	0.6	0.0	2.2	0.7	3.7	0.6	11.3	1.9
Bulgaria	466	10.3	13.9	0.2	0.5	75.1	0.0	0.0	0.1	0.9
Croatia	5309	3.2	9.0	0.0	0.1	87.6	0.3	0.4	1.2	0.5
Cyprus	641	3.7	6.6	0.7	1.8	87.1	0.0	0.0	0.0	0.2
Denmark	6527	12.2	61.0	0.3	1.2	25.4	11.5	2.9	33.6	6.6
Estonia	2559	0.0	48.9	0.5	0.0	50.6	0.1	0.3	2.9	0.0
Faroe Islands	919	1.1	15.3	0.0	0.0	83.6	0.2	–	0.5	0.4
Finland	17922	3.3	27.8	0.1	3.1	65.8	5.1	1.7	14.6	3.3
France	8942	20.8	29.7	0.3	8.2	41.0	19.7	0.7	52.4	22.6
Germany	4952	46.5	37.2	0.3	0.1	15.9	32.3	0.7	87.1	31.9
Gibraltar	14	29.9	0.0	0.0	0.0	70.1	0.0	–	0.1	0.0
Greece	14267	7.2	7.4	0.0	0.7	84.6	0.2	0.1	1.6	7.3
Iceland	7971	0.6	36.4	0.4	0.1	62.4	5.1	16.5	5.7	24.2
Ireland	6630	3.9	38.5	0.2	1.7	55.8	5.0	0.9	21.4	1.6
Italy	8813	22.1	17.8	0.1	0.7	59.2	9.9	0.4	25.6	18.5
Latvia	684	16.8	47.5	0.7	10.6	24.4	2.5	5.6	4.5	1.4
Lithuania	356	42.1	45.4	0.0	0.0	12.5	1.5	1.9	3.4	0.8
Malta	190	19.1	5.5	0.0	0.0	75.4	0.0	0.1	0.1	0.9
Monaco	5	33.1	0.0	0.0	0.0	66.9	0.0	–	0.0	0.1
Montenegro	395	3.1	17.0	0.0	0.7	79.2	0.0	0.1	0.1	0.2
Netherlands	4523	75.2	14.7	0.4	0.1	9.6	59.9	5.4	132.4	867.0
Norway	52984	1.3	13.9	0.1	0.2	84.5	26.4	5.4	30.2	153.8
Poland	1507	38.5	37.3	1.5	7.6	15.2	9.1	1.1	16.5	8.2
Portugal	3273	2.6	31.4	0.3	7.4	58.4	1.3	0.5	3.5	0.3
Romania	1084	4.0	67.5	0.1	0.2	28.1	0.1	0.0	0.3	2.1
Slovenia	53	45.7	6.0	0.0	1.4	46.9	0.0	0.1	0.1	0.4
Spain	7648	14.1	16.4	0.1	2.2	67.1	3.2	0.2	10.2	4.9
Sweden	24115	1.9	20.4	0.3	1.1	76.3	7.5	1.3	11.6	14.3
Turkey	827	1.6	5.4	4.1	0.3	88.7	0.0	0.0	0.0	2.0
Ukraine	1732	0.0	22.0	0.9	0.1	77.0	0.1	0.1	0.1	0.0
United Kingdom	19867	9.8	24.1	0.1	3.6	62.3	37.6	1.1	125.1	44.0
Total	214891	8.7	21.8	0.2	1.6	67.8	243.3	1.1	597.8	1246.2

Costs and damages are given in 2024 values and expressed as a percentage of each country’s GDP for 2023^44^. Only the European coastlines of Russia and Turkey are reported.

Reported costs include adaptation costs in 2020 (absolute values and as a percentage of 2023 GDP) and the total discounted costs (discount rate = 3%) of adaptation costs and residual expected flood damages over the full time horizon (2020-2150). Results are shown per country and for Europe as a whole under the SSP2-4.5 scenario.

In Belgium, the Netherlands, and Germany, protection is implemented along 97%, 75%, and 47% of their total coastline in 2150, respectively (Table 1). This reflects the combination of high extreme sea levels in the North Sea and low-lying coastal areas with a high concentration of assets. Conversely, protection is economically optimal for only 3% or less of the total coastline in Åland, Estonia, the Faroe Islands, Iceland, Norway, Portugal, Sweden, Turkey and Ukraine. These countries feature extensive steep coastlines where no adaptation is required, and in areas where coastal floodplains necessitate adaptation, accommodation or retreat is more economically viable than protection due to the low asset values.

### Where and when do adaptation tipping points occur?

**Fig. 4:**
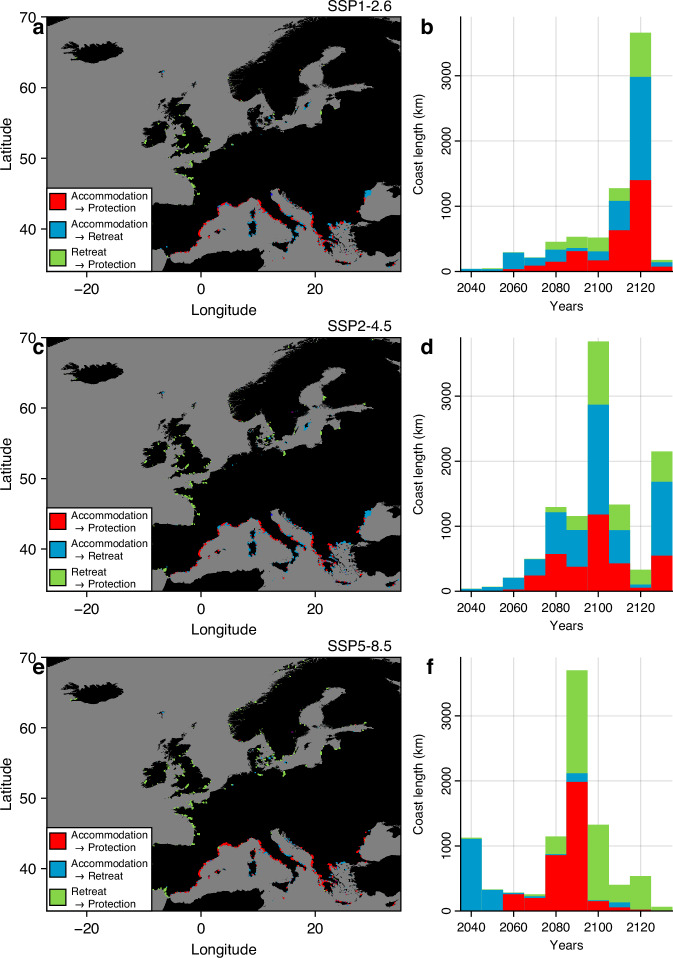
The geographic location and timing of adaptation tipping points. **a**,**c**,**e** Maps showing the geographic location of floodplains where certain ATP occur under **a** SSP1-2.6, **c** SSP2-4.5 and **e** SSP5-8.5. **b**,**d**,**f** Stacked bar charts illustrating the timing of ATP (x-axis) for the accumulated length of coastline (y-axis) for all floodplains under **b** SSP1-2.6, **d** SSP2-4.5 and **f** SSP5-8.5, using the same color codes as in (**a**), (**c**), and (**e**). Eurostat (GISCO), 2024. Licensed under CC BY 4.0: https://creativecommons.org/licenses/by/4.0/ⓒEuroGeographics for administrative boundaries in the basemaps.

The majority of ATP occur along the Mediterranean and Black Sea coasts (Fig. 4), where extreme sea levels and current flood protection levels are lower compared to the North Sea or Baltic Sea (e.g., breakwaters, ripraps, and small walls rather than higher dikes). Floodplains in the Mediterranean and Black Sea can adapt to SLR by accommodation until they reach ATP towards the beginning of the next century under SSP1-2.6 (Fig. 4a, b) and toward the middle or end of this century under SSP5-8.5 (Fig. 4e, f). Once these tipping points are reached, options effective under higher SLR, such as protection or retreat, will become necessary, with protection being the optimal choice in areas of higher asset concentration. The length of the European coastline that does not reach these tipping points until 2150 is five times as long under SSP1-2.6 compared to SSP5-8.5. If these ATP are not reached, accommodation can continue, particularly along the coastlines of Bulgaria and Turkey, where extreme sea levels increase little with increasing return periods (Fig. 3 and Table S1 and S2).

Further ATP arise in Scandinavia, the UK, and along the Atlantic coasts of Portugal, Spain, and France, necessitating protection along previously retreated coastlines (Fig. 4e, f). In these areas, future high SLR will increase flood risks for communities situated at higher elevations that were not previously protected in previous time steps. Consequently, additional protection, implemented inland at higher elevation levels, becomes the economically optimal adaptation option to safeguard the remaining residents and assets in these regions (example given in Figure S4).

**Fig. 5:**
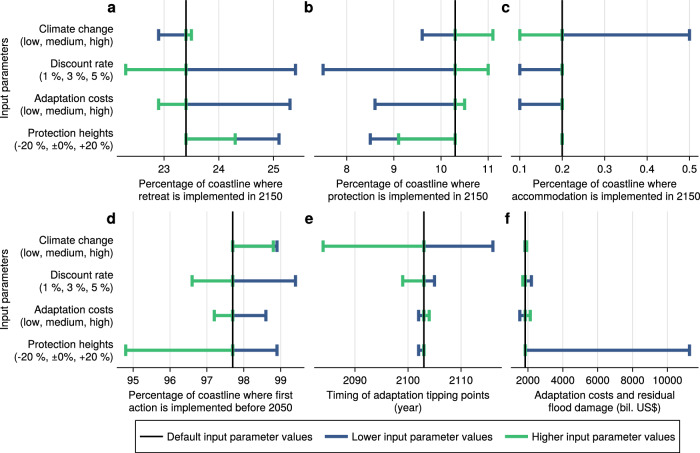
One-factor-at-a-time sensitivity analysis. **a–f** The bar charts illustrate how variations in input parameters influence our results for the entire European coastline, considering the following metrics: **a** retreated coastline in 2150, **b** protected coastline in 2150, **c** accommodated coastline in 2150, **d** floodplains implementing their first adaptation action before 2050, **e** the timing of adaptation tipping points, and **f** the net present value of costs. The default input parameters are the SSP2-4.5 climate change scenario, a 3% discount rate, medium adaptation costs for protection and retreat, and medium initial protection heights. Lower input parameters, shown in blue, correspond to SSP1-2.6, a discount rate of 1%, low adaptation costs for protection and retreat, and a reduction of initial protection heights by 20%. Higher input parameters, shown in green, correspond to SSP5-8.5, a discount rate of 5%, high adaptation costs for protection and retreat, and an increase of initial protection heights by 20%.

Different climate change scenarios substantially impact the timing of ATP. Under SSP1-2.6, ATP predominantly occur in the early 22nd century, while under SSP2-4.5, this transition occurs primarily in the late 21st century. In contrast, under SSP5-8.5, ATP emerge as early as the mid-21st century (Fig. 4). On average, ATP occur 32 years earlier under SSP5-8.5 compared to SSP1-2.6 (see section on sensitivity analysis and Fig. 5).

The occurrence of ATP can be completely attributed to SLR, because ATP practically does not occur in a counterfactual scenario without SLR (Figs.S1, 3). Comparing a counterfactual scenario without SED to SSP2-4.5 (Fig.S1) further illustrates that SED influences the type of ATP. Without SED, only very few switches from accommodation to protection occur because lower asset values and reduced EAD do not justify protection along the same coastal stretches. In the absence of SLR, SED alone is insufficient to trigger any ATP in our model. Although asset growth under SED increases EAD, it does not raise the benefit-cost ratios of protection or retreat above those of accommodation; this is achieved only through SLR, which directly increases the hazard component and therefore raises EAD.

In contrast to climate change scenarios, parameters, such as the discount rate, adaptation costs, and initial protection levels, have almost no impact on the timing of ATP (Fig. 5). This highlights that the timing of ATP is predominantly driven by climate change scenarios, and also that these results are robust despite uncertainties in socioeconomic parameters.

### Costs for adaptation and residual flood damage

The net present value of costs, defined here as the discounted sum of adaptation cost and residual expected annual flood damages (EAD) over the full time horizon under economically optimal adaptation pathways, is US$ 1.8 trillion under SSP2-4.5 (all costs are reported in 2024 US$), with adaptation costs accounting for 32% (Table 1). This represents 8% of the combined GDP of all the considered countries in 2023. Notably, almost half of the adaptation investments over the entire time horizon are concentrated in the first time step (Fig. 2, note that this concentration is less apparent due to the logarithmic y-axis scale). Although retreat is implemented more than two times more frequently than protection along the length of the European coastline (Fig. 3), the adaptation costs for protection exceed those for retreat (Fig. 2). This is because retreat costs depend on the value of assets in the areas being retreated, making retreat from larger sections of the coastline with low asset values cheaper than implementing protection in a few high-asset areas.

EAD start at US$ 121 billion in 2020 but declines substantially to US$ 0.8 billion in 2030 after initial adaptation investments are made in 2020, reflecting that Europe is substantially under-protected in the base year (Figure 2). Over the first decade (2020-2030), EAD accumulates to US$ 1,210 billion (5.4% of Europe’s GDP in 2023), far exceeding the one-time adaptation investments of US$ 243 billion in 2020 (1.1% of Europe’s GDP in 2023)^44^. After this initial adjustment, adaptation needs fall substantially and over much of the subsequent time horizon, adaptation costs surpass residual damages, even when EAD is considered over full ten-year time steps (Figure 2). Towards the end of the time horizon, EAD rises again because adaptation investments to maintain protection levels cease after 2130; with only two decades remaining until 2150, further protection upgrades are no longer cost-effective, leading to increasing residual flood damages under rising sea levels. Regularly updating economic assessments with appropriate time horizons and additional information is crucial to ensure accuracy and relevance over time.

### Sensitivity Analysis

A sensitivity analysis reveals that variations in socioeconomic parameters, particularly initial protection heights, have the greatest influence on the net present value of costs, whereas the impact of climate change is negligible (Fig. 5), aligning with recent findings^32,45^. The net present value of costs for Europe ranges between US$ 1.83-1.93 trillion under different climate change scenarios (Table S1 and S2). The reason for this small cost difference despite their large difference in SLR lies in the fact that large investments need to be made in the first time step, with the next round of investments is to some extent discounted away as it only occurs in the mid to late-century (Fig. 2). For example, while the initial adaptation costs in 2020 are only 5% higher under SSP5-8.5 compared to SSP1-2.6, adaptation costs in the mid to late century are more than twice as high under SSP5-8.5 as compared to SSP1-2.6 (Fig. 2). The latter increase is driven by a fourfold increase in the length of the European coastline crossing ATP towards protection in the mid to late century under SSP5-8.5 compared to SSP1-2.6 (Fig. 4). In contrast, variations in socioeconomic parameters, such as low initial protection heights and a low discount rate, increase the total discounted cost for Europe by 611% and 19%, respectively (Fig. 5).

## Discussion

The future EAD attained here is substantially lower than that of previous European coastal flood risk assessments, which is as expected, because previous European assessments overestimate EAD due to the limitation described in the Introduction, in particular the consideration of a constant single adaptation option (i.e., protection). For example, Europe’s EAD in 2100 is estimated by Vousdoukas et al.^29^ to be US$15 billion under SSP1-4.5 and US$42 billion under SSP5-8.5, and by Lincke and Hinkel^28^ to approximate US$13 billion under SSP1-2.6 (converted to 2024 US$). Our projected EAD in 2100 is only 5% of the value reported by ref. ^29^ under SSP5-8.5 and 13% under SSP2-4.5 (Fig. 2). The lower EAD can be explained by two reasons. First, single-stage decision making used in ref. ^29^ leads to under-adaptation towards the end of the time horizon considered, and, consequently, overestimates EAD. Second, considering multiple options (protection, retreat, and accommodation) can more efficiently reduce flood damages, because this allows for better tailoring the type of response to the diverse biophysical and socio-economic characteristics of each floodplain.

For similar reasons, the length of Europe’s protected coastline in our study (i.e., 9%) is substantially lower than the 23% - 32% suggested by ref. ^29^, which considered only protection. A recent study^27^ that considered both protection and retreat closely aligns with our findings, estimating that 9% of Europe’s coastline should be protected across all scenarios. This alignment underscores a key insight: the length of the economically optimally protected coastline is lower when retreat is considered as an alternative.

Our study has several limitations. The range of adaptation options considered is limited; for example, accommodation for higher sea levels or advance (land claim or raising) are not included, which could delay adaptation tipping points or enable new pathways. Socio-economic scenarios are represented only at broad scales, potentially overlooking local dynamics, such as population growth in low-density coastal areas, which could increase retreat costs and residual flood damages. We focus solely on coastal flooding, whereas other related hazards, such as coastal erosion, heavy precipitation, and riverine flooding, are not considered. These hazards could require additional adaptation investments; for instance, global dike investments for riverine flooding could increase our adaptation costs in the first time step by roughly 36%^46^. Other hazards may also interact with the adaptation options considered here - for example, coastal protection could exacerbate erosion or impede drainage during heavy precipitation. In our model, retreat is implemented in the first time step for 99% of floodplains. While this approach may prevent new coastal developments that could lock floodplains into a protection pathway, potential coastal development opportunities, such as ports, are not represented in the model. Finally, our analysis focuses exclusively on economic optimality, neglecting ecological, technical, societal, legal, and political dimensions of adaptation.

The qualitative expert-judgment-based work on generic adaptation pathways^7,10,11^ predicts ATP from accommodation to either protection or retreat, but none from protection to retreat, which can be verified by our results (Fig. 4). The generic pathways recommend immediate implementation of no-build zones in order to keep the option of retreat more open (and cheaper) in the future, aligning with our results, where retreat is almost always implemented immediately. Additionally, case-study-based conclusions from the IPCC Special Report in the Ocean and Cryosphere in a Changing Climate (SROCC)^47^ and AR6^7^ suggest that accommodation is a highly effective adaptation option for small amounts of SLR, allowing time to prepare for larger changes, which is verified by our results. Quantitatively, this is, however, only the case for the Mediterranean and Black Sea coast (Fig. 4), where extreme sea levels are lower than those along the Atlantic and Baltic coastlines.

In contrast to the generic adaptation pathways, in which retreat occurs as the “last resort” implemented over time^10^, our results show that retreat is sometimes followed by, and combined with, protection. Specifically, for sections of the coastlines in Portugal, France, the UK, Sweden, and Norway, our analysis suggests an initial retreat to approximately 1-3 meters above sea level where relatively undeveloped land exists along the coast. By the mid-21st century, however, ATP for this initial retreat is reached, and new inland protection for the remaining settlements is implemented (Figs. 4 and S4).

In contrast to our findings, which indicate that retreat is economically optimal for almost one-fourth of Europe’s coastline, empirical evidence suggests that protection remains the prevailing coastal adaptation option in Europe, although an increasing number of adaptation approaches now combine it with accommodation, retreat and protection through nature-based solutions^6,48^. Although accommodation is becoming more common in certain parts of the European coastline, it is still poorly integrated into overarching adaptation policies^49^, and more detailed analyses suggest protection of many less developed coasts will be hard to sustain to 2100^50,51^. Our large-scale economic analysis provides a strong justification for integrating accommodation and retreat into overarching adaptation policies.

## Methods

**Fig. 6:**
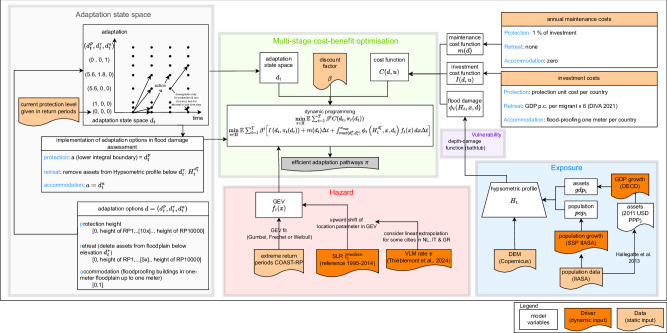
Workflow chart: a detailed visualization of the paper’s methodology. From Data Input and Preprocessing in the Exposure component, Hazard component, Cost Functions, and Adaptation State Space to the Cost-Benefit Optimization.

Our multi-stage, sometimes referred to as dynamic, cost-benefit optimization integrates several components: A hazard component to model extreme sea level, an exposure component to assess population and assets at risk, a vulnerability component to assess the susceptibility of assets to hazards, an adaptation state space to outline potential adaptation pathways, and cost functions to estimate the costs associated with adaptation actions (Fig. 6). The multi-stage cost-benefit optimization is conducted for each coastal floodplain individually.

We consider three different coastal adaptation options against coastal flooding: i) protection using hard protection infrastructure, such as dikes, ii) accommodation by flood-proofing buildings located within the one-meter floodplain up to the first meter of their height, and iii) (planned) retreat from the coast.

### Adaptation state space

Let ${d}_{t}=({d}_{t}^{p},{d}_{t}^{r},{d}_{t}^{a})$ represent the adaptation state space over time, where ${d}_{t}^{p}$ represents the protection height, ${d}_{t}^{r}$ represents the retreat height, and ${d}_{t}^{a}$ represents accommodation. Protection is given in absolute protection heights above mean sea level in meters (10 values ranging from the 1 year extreme event height to the 1 in 10,000 year extreme event height + SLR in 2150), retreat is given in absolute elevation heights in meters below which all assets and people will retreat (5 values ranging from the 1 year extreme event height to the 1 in 10,000 year extreme event height + SLR in 2150), and accommodation is binary (${d}_{t}^{a}\in \{0,1\}$), indicating whether all buildings in the one-meter floodplain are flood-proofed up to one meter. We limit accommodation to 1 meter, as reported European cases do not exceed this level^52^. The adaptation action *u* = (*u*^*p*^, *u*^*r*^, *u*^*a*^) can increase each adaptation option independently of each other by

1 $${d}_{t+1}={d}_{t}+u=({d}_{t}^{p}+{u}^{p},{d}_{t}^{r}+{u}^{r},{d}_{t}^{a}+{u}^{a}),\,{u}^{r}\geqq 0,$$

where the protection height and accommodation can be decreased to zero. The state space allows combinations of protection and retreat at the same time if the protection height is higher than the retreat height. The following ATP types are considered in the adaptation state space over time: switching from protection to accommodation, retreat or protection and retreat; switching from accommodation to protection, retreat or protection and retreat; and switching from retreat to protection and retreat.

### Costs of adaptation options

We model country-specific unit costs for coastal protection using a linear cost function, with a unit cost *C* representing the expense for a one-meter dike increment per kilometer of length. For each country, the base unit cost of dikes in the Netherlands, ranging from €4.5M to €12.4M (2009 values) for rural areas^53^, is adjusted using a country-specific cost factor (*CCF*_*i*_), similar to previous studies^54–57^. The unit cost estimates for the Netherlands are converted to 2011 USD PPP by applying GDP deflator values for 2009 - 2011 and a 2011 purchasing power parity (PPP) conversion factor, sourced from the World Bank (https://data.worldbank.org/indicator/NY.GDP.DEFL.ZS?locations=USand https://data.worldbank.org/indicator/PA.NUS.PPP?locations=NL). Using these adjusted values, low and high unit cost estimates for other countries are calculated based on the rural low and high cost estimates from the Netherlands:

2 $${C}_{i,low}=CC{F}_{i}*{C}_{NLD,low}$$

3 $${C}_{i,high}=CC{F}_{i}*{C}_{NLD,high}$$

The resulting unit protection costs for all European countries are listed in Table 2.

**Table 2:** Protection unit costs per country in million USD per meter dike height and kilometer dike length (2011 PPP values)

Country	Low	Mean	High
Aaland	2.7	5.1	7.5
Albania	2.7	5.1	7.5
Belgium	5.4	10.2	15.0
Bosnia and Herzegovia	1.6	3.1	4.5
Bulgaria	2.7	5.1	7.5
Croatia	1.6	3.1	4.5
Cyprus	2.7	5.1	7.5
Denmark	5.4	10.2	15.0
Estonia	5.4	10.2	15.0
Faroe Islands	5.4	10.2	15.0
Finland	2.7	5.1	7.5
France	3.8	7.1	10.5
Germany	5.4	10.2	15.0
Gibraltar	6.0	11.2	16.5
Greece	2.2	4.1	6.0
Iceland	4.9	9.2	13.5
Ireland	6.0	11.2	16.5
Italy	3.8	7.1	10.5
Latvia	5.4	10.2	15.0
Lithuania	5.4	10.2	15.0
Malta	2.7	5.1	7.5
Moldova	5.4	10.2	15.0
Monaco	1.1	2.0	3.0
Montenegro	2.7	5.1	7.5
Morocco	4.3	8.2	12.0
Netherlands	5.4	10.2	15.0
Norway	3.3	6.1	9.0
Poland	5.4	10.2	15.0
Portugal	2.2	4.1	6.0
Romania	5.4	10.2	15.0
Russia	5.4	10.2	15.0
Slovenia	1.6	3.1	4.5
Spain	2.7	5.1	7.5
Sweden	2.7	5.1	7.5
Turkey	2.7	5.1	7.5
Ukraine	5.4	10.2	15.0
United Kingdom	6.0	11.2	16.5

We convert these costs from US$ 2011 to US$ 2024 using the GDP deflator values sourced from the World Bank (https://data.worldbank.org/indicator/NY.GDP.DEFL.ZS?locations=US). We also assume a fixed cost component for protection, which makes small upgrades relatively more expensive than large upgrades, which is consistent with other studies^40,58^:

4 $$I(d,u)=0.3*c*1*l+0.7*c*u*l,$$

where *u* is the height upgrade, *c* the unit cost factor, and *l* the length of the protection action. Similar to previous studies^52,59,60^, we assume maintenance costs for coastal protection infrastructure to be 1% of initial investment costs. We note that the model does not include spatial constraints on dike construction or upgrading, nor the costs of breaching or possibly dismantling dikes when shifting from protection to retreat. Both factors could substantially increase adaptation costs, especially when switching between options, as demonstrated in a recent study^61^.

In line with prior research^27^, we estimate retreat costs at six times the local GDP per capita per migrant, assuming no maintenance costs for the retreat.

For accommodation, we use average unit costs for flood-proofing buildings, derived from rescaled data from a UK case study reviewed in ref. ^52^. We convert these costs to a per-person per-meter basis using the average UK household size in 2008 (2.36 persons per household;^62^). The resulting estimated average unit cost for flood-proofing buildings is US$8,643 (2016) per person per meter (Table 3). This value is then adjusted using the country-specific cost factors (*CCF*_*i*_) and converted to 2024 USD using the World Bank GDP deflator^63^. Maintenance costs for accommodation are assumed to be zero.

**Table 3:** Accommodation unit costs (in 2016 US $) for flood-proofing buildings per case study, adapted from Aerts, J. C. J. H. (2018)

Case study	Year	Height (m)	Average cost	Average cost per person	Average cost per person per meter
USA	2009	0.6	12,326	4,814	8,023
USA	2009	2.0	18,900	7,383	3,692
United Kingdom	2008	0.9	18,359	7,779	8,643

A Review of Cost Estimates for Flood Adaptation. Water, 10(11), 1646. 10.3390/w10111646published under the license https://creativecommons.org/licenses/by/4.0/^52^.

### Current protection levels

We use estimates of current coastal flood protection levels at the NUTS2 level, derived from a European survey^64^ distributed to coastal experts across Europe. This survey represents the only systematic empirical evidence of coastal flood protection levels at a continental scale^37,54^. When a range of protection levels is reported for a NUTS2 unit, we differentiate protection levels based on population density (people per square kilometer), categorizing areas as uninhabited ( < 10), sparsely populated ( < 300), or densely populated ( > 300). For regions where the survey results do not provide data on current protection levels, we supplement with modeled estimates^31^ and assume no protection where such estimates are unavailable.

### Exposure and vulnerability using DIVACoast

We follow the general approach of the DIVA framework^20^, which has been widely applied to assess flood damages at continental and global scales^17,20–23,26–28^. To improve this prior work, now called DIVACoast, we increase the scale of analysis and divide the European coastal zone into 41,327 coastal floodplains^65^, compared to the 1,810 segments in the original DIVA model. These floodplains are defined as hydrologically connected regions below the 1-in-100 year flood level, with an additional 2-meter allowance for SLR, and are further divided by NUTS2 administrative units as required. The floodplains are derived from the Copernicus digital elevation model (DEM)^66^ and extreme water level data from COAST-RP^67^.

For each coastal floodplain, we generate a hypsometric profile *H* by overlaying the copernicus DEM^66^ with population data from the Global Human Settlement Layer^68^, following the methodology outlined in ref. ^69^. In order to correct for different vertical datums all digital elevation data is converted to be referenced to GOCO06s. To do so, conversion grids from GFZ have been used (https://icgem.gfz.de/calcgrid). In order to align the digital elevation data with the surge data, mean dynamic topography data has been used^70^. Asset exposure is calculated by multiplying local GDP per capita^71^ by a factor of 2.8^19^ and the population data. Future asset exposure is determined by applying population and GDP growth rates from the SSP scenarios^72^ to the asset exposure until 2100, with linear trend extrapolations applied from 2100 onwards. In the case of retreat, we modify the hypsometric profile by removing all assets and people below a certain elevation ${d}_{t}^{r}$, resulting in ${H}_{t}^{{d}_{t}^{r}}$.

We calculate the flood damage *ϕ*_*t*_(*H*_*t*_, *x*, *d*) on the hypsometric profile *H*_*t*_ under extreme water level *x* and adaptation state *d* using a bathtub flood model and a logarithmic depth damage function $\frac{w}{w+1}$ for inundation depth *w*, following the approach in^20^.

### Hazards

We rely on regional mean sea-level projections ${l}_{t}^{p}$ provided by the 6th Assessment Report of the IPCC^73^. Note that we remove the low-confidence vertical land motion (VLM) estimates of the AR6^74^ and replace them with Glacial Isostatic Adjustment (GIA) model results^75^. In this work, we consider the median (*p* = 0.5) of the medium confidence projections for SSP1-2.6, SSP2-4.5, and SSP5-8.5.

For hotspot regions in Europe where substantial vertical land motion (usually subsidence) beside GIA have been observed, are robust, and are still recorded (i.e. north-east Netherlands, north-west Germany, north Italian Adriatic coast and in the vicinity of Thessaloniki in Greece), we assume a linear extrapolation of vertical land motion contribution based on the Copernicus European Ground Motion Service (EGMS) vertical velocity estimates from the Ortho product for the period 2015 - 2021^76^. More specifically, we derive the spatially averaged EGMS vertical land velocity every 1 km along the shoreline of the hotspot regions. The spatial average is calculated over a 10 km-radius area around each 1 km-spaced shoreline point in order to smooth out fine-scale VLM variations. Following^77^, the VLM estimates are then adjusted to the geocentric reference frame ITRF2014 to avoid an overestimation of the subsidence. We also subtract the GIA contribution, which is already included in the mean sea-level projections. Finally, the obtained VLM *v*_*t*_ are extrapolated linearly for the entire time horizon and added to coastal sea-level projections to determine the relative sea-level rise. Overall, this leads to a near 2 mm/yr relative SLR enhancement in the hotspot regions and up to 4 mm/yr locally for some coastal floodplains of the north Italian Adriatic coast.

Extreme water level distributions are derived by fitting Generalized Extreme Value (GEV) and Generalized Pareto (GPD) distributions to the extreme water level return periods provided by COAST-RP^67^. The best-fitting distribution is selected based on least-squares optimization. These return periods are available at a high resolution of 1.25 km along the European coastline. To associate each coastal floodplain with its corresponding extreme water level return period, we perform a nearest-neighbor matching based on the midpoint of the coastline for each floodplain. This process produces an extreme value distribution with a corresponding probability distribution function *f*(*x*) for each location. Extreme water level distributions are modified over time by SLR ${l}_{t}^{p}$ and vertical land motion *v*_*t*_. We implement this by shifting the location parameter *μ* of the extreme value distribution function upwards with $\mu+{l}_{t}^{p}-{v}_{t}$, resulting in the probability distribution function *f*_*t*_(*x*).

### Multi-stage cost-benefit optimization

We apply a multi-stage cost-benefit optimization for each coastal floodplain individually and minimize the expected total discounted costs over time by choosing the optimal adaptation pathway *π*, which defines which adaptation action *u* should be taken at the adaptation state *d*_*t*_,

5 $$\,{{\min }}_{\pi \in \Pi} \,{\sum }_{{t}_{0}}^{T}{\beta }^{t}C({d}_{t},{\pi }_{t}({d}_{t})),$$

where *β* is the discount rate and *C*(*d*, *u*) is the cost function. Let *I*(*d*, *u*) be the investment cost function and *m*(*d*) the annual maintenance cost function (see Section on Costs of adaptation options for details). Using the flood damage function *ϕ*_*t*_(*H*_*t*_, *x*, *d*) we can then write our objective function (5) as

6 $$\,{{\min }}_{\pi \in \Pi} \,{\sum }_{{t}_{0}}^{T}{\beta }^{t}\left[I\left({d}_{t},{\pi }_{t}({d}_{t})\right)+m({d}_{t})\Delta t+{\int }_{max({d}_{t}^{p},{d}_{t}^{a})}^{\infty }{\phi }_{t}\left({H}_{t}^{{d}_{t}^{r}},x,{d}_{t}\right){f}_{t}(x)\,dx\Delta t\right],$$

where we integrate over the flood damages multiplied by the probability distribution function of the extreme water levels *f*_*t*_(*x*) to consider the expected annual flood damages times Δ*t*, the number of years between two time steps. The lower bound of the integral is set to $max({d}_{t}^{p},{d}_{t}^{a})$, because we assume that for extreme water levels below the protection height or the accommodation height, no flood damage occurs. This also means that as soon as the protection or accommodation height is exceeded by an extreme water level, flood damage is considered as if there were no adaptation action in place, i.e., a complete failure of coastal protection or accommodation.

We set (*t*_0_, Δ*t*, *T*) to (2020, 10, 2150) and solve the objective function (6), which is in the form of a Bellman equation^78^, using dynamic programming^79^. We assume a discount rate of 3%, consistent with the average values used in similar studies^27–29^ and in line with the European Commission’s 2024 recommendations for discount rates.

### Sensitivity analysis

For the sensitivity analysis, we conduct 11 additional simulations with varied input parameters. The default input parameters are based on the SSP2-4.5 climate change scenario, a 3% discount rate, medium adaptation costs and medium initial protection heights. We assess 11 other simulations by adjusting the climate change scenario to SSP1-2.6 and SSP5-8.5, representing a low and very high greenhouse gas emission scenario to span a wide range of possible futures. We adjust the discount rate from 3% to both 1% and 5%, similar to discount ranges considered in sensitivity analyses in other studies^27,28^ and also spanning the range of ongoing discussion about discount rates between low discount rate (1.4%^80^) and high discount rates (4.3%^81^). We adjust the medium protection costs to low and high estimates, as this range was identified when deriving protection costs. We also adjust the medium retreat costs, calculated as six times the local GDP per capita per migrant, to 2 (low) and 10 (high), which spans the uncertainty range reported in ref. ^27^. We vary initial protection heights by assuming reductions of 20% and increases of 20% on the absolute heights. We also perform counterfactual simulations using the default input parameters under three conditions: without SLR, without SED, and without both SLR and SED.

## Supplementary information

Supplementary Information

Transparent Peer Review file

## Data Availability

Additional results of this study are provided in the Supplementary Information. The input data, processed data and data that was used to conduct the results of this research can be found under 10.5281/zenodo.19484283. Map plots were generated using country-level geospatial data from Eurostat (GISCO), which are made available under the Creative Commons Attribution 4.0 International (CC BY 4.0) licence and were downloaded from https://ec.europa.eu/eurostat/web/gisco/geodata/administrative-units/countries: ⓒEuroGeographics for the administrative boundaries.

## Code availability

The applied code for the DIVACoast model is available under https://gitlab.com/globalclimateforum/DIVACoast.jland for the dynamic programming of the multi-stage cost-benefit analysis under https://gitlab.com/globalclimateforum/AdaptiveDM. The code that was used to conduct the results of this research can be found under 10.5281/zenodo.19484283.
