# Supplementary material for: The economically optimal mix and timing of coastal adaptation in Europe to 2150
Source: Nat Commun. 2026 Jul 15;17:6249. doi: 10.1038/s41467-026-74042-8 (PMC13373216; doi:10.1038/s41467-026-74042-8)
Supplement: Supplementary file 1 — Supplementary Information [file 41467_2026_74042_MOESM1_ESM.pdf]

Supplementary Information: The economically optimal mix and timing of coastal adaptation in Europe to 2150

Vanessa Völz[1,2*], Jochen Hinkel[1,2], Daniel Lincke[2], Lars E. Honsel[2], Robert J. Nicholls[3], Rémi Thiéblemont[4], Gonéri Le Cozannet[4], Paul Sayers[5]

[1*]Thaer-Institute of Agricultural and Horticultural Sciences, Humboldt-Universität zu Berlin, Unter den Linden 6, 10099, Berlin, Germany.
[2]Global Climate Forum, Neue Promenade 6, 10178, Berlin, Germany.
[3] Tyndall Centre for Climate Change Research, University of East Anglia (UEA), Norwich, NR4 7TJ, UK.
[4] BRGM - French Geological Survey, 3 avenue de la Claude Guillemin, Orléans, 45100, Loiret, France.
[5] Sayers and Partners, High Street, Watlington, OX49 5PY, Oxfordshire, UK.

*Corresponding author(s). E-mail(s): vanessa.voelz@globalclimateforum.org;

Appendix A

This supplementary information contains additional results on low and high climate change scenarios (Table S1 and S2), counterfactual scenarios without sea-level rise (SLR) and/or socio-economic development (SED) (Figure S1, S2, S3) and a local example where an ATP from retreat to protection occurs (Figure S4).

Table S1: The proportion of the coastline for which different adaptation options are implemented in 2150, along with the net present value of costs. Reported costs include adaptation costs in 2020 (absolute values and as a percentage of 2023 GDP) and the total discounted costs (discount rate = 3%) of adaptation costs and residual expected flood damages over the full time horizon (2020–2150). Results are shown per country and for Europe as a whole under the SSP1-2.6 scenario.

Country	Coast length (km)	Protection (%)	Retreat (%)	Accommodation (%)	Protect and retreat (%)	Nothing (%)	Adaptation costs in 2020 (bil. US$)	Adaptation costs in 2020 (% of 2023 GDP)	Total adaptation costs (bil. US$)	Total residual flood damage (bil. US$)
Åland	3886	0.0	3.2	2.1	0.0	94.7	0.0	-	0.2	0.0
Albania	753	43.6	10.7	0.1	0.0	45.5	0.7	3.0	1.1	25.5
Belgium	319	96.5	0.9	0.0	1.9	0.7	3.1	0.5	10.8	2.1
Bulgaria	466	1.2	0.0	23.7	0.0	75.1	0.0	0.0	0.0	0.9
Croatia	5309	3.6	8.6	0.0	0.2	87.6	0.3	0.4	1.1	0.5
Cyprus	641	3.9	7.2	1.0	0.8	87.1	0.0	0.0	0.0	0.2
Denmark	6527	12.3	61.4	0.2	0.5	25.7	10.9	2.7	32.1	6.2
Estonia	2559	0.0	47.9	0.3	0.0	51.8	0.1	0.3	2.9	0.0
Faroe Islands	919	1.1	15.3	0.0	0.0	83.6	0.2	-	0.5	0.4
Finland	17922	1.8	31.9	0.2	0.3	65.8	6.5	2.2	21.3	3.8
France	8942	21.8	30.1	0.2	6.7	41.3	18.7	0.6	50.3	22.3
Germany	4952	46.6	37.0	0.3	0.1	16.0	31.8	0.7	84.7	31.9
Gibraltar	14	29.9	0.0	0.0	0.0	70.1	0.0	-	0.1	0.0
Greece	14267	7.9	6.1	1.2	0.4	84.5	0.2	0.1	1.2	7.3
Iceland	7971	0.7	36.4	0.3	0.1	62.5	5.1	16.5	5.7	24.4
Ireland	6630	3.9	39.3	0.1	0.8	55.9	4.9	0.9	21.2	1.4
Italy	8813	22.9	16.7	0.3	0.6	59.6	10.3	0.5	24.1	18.6
Latvia	684	16.8	51.0	0.3	6.7	25.1	2.2	5.0	4.2	1.6
Lithuania	356	42.1	45.4	0.0	0.0	12.5	0.3	0.4	2.8	1.2
Malta	190	19.4	5.2	0.0	0.0	75.4	0.0	0.1	0.0	0.9
Monaco	5	33.1	0.0	0.0	0.0	66.9	0.0	-	0.0	0.1
Montenegro	395	3.1	17.3	0.0	0.4	79.2	0.0	0.1	0.0	0.3
Netherlands	4523	75.9	12.9	0.5	0.8	9.9	57.2	5.1	128.8	866.4
Norway	52984	1.3	14.0	0.1	0.1	84.5	26.0	5.4	29.3	154.0
Poland	1507	41.3	41.8	0.1	0.1	16.7	9.4	1.2	16.1	7.6
Portugal	3273	2.6	34.2	0.1	4.5	58.6	1.3	0.4	3.4	0.3
Romania	1084	4.5	64.8	2.6	0.1	28.0	0.1	0.0	0.3	2.0
Slovenia	53	32.6	6.0	0.0	14.5	46.9	0.0	0.1	0.3	0.4
Spain	7648	14.6	17.1	0.1	0.9	67.3	3.0	0.2	9.5	4.3
Sweden	24115	1.7	20.2	1.1	0.5	76.5	7.3	1.2	11.1	15.3
Turkey	827	0.5	2.6	8.2	0.0	88.7	0.0	0.0	0.0	2.0
Ukraine	1732	0.0	17.6	1.2	0.0	81.2	0.1	0.0	0.1	0.0
United Kingdom	19867	9.7	25.0	0.1	2.8	62.3	39.9	1.2	124.1	43.2
Total	214891	8.7	22.0	0.5	0.9	67.9	239.9	1.1	587.5	1245.1

Costs and damages are given in 2024 values and expressed as a percentage of each country's GDP for 2023. Only the European coastlines of Russia and Turkey are reported.

Table S2: The proportion of the coastline for which different adaptation options are implemented in 2150, along with the net present value of costs. Reported costs include adaptation costs in 2020 (absolute values and as a percentage of 2023 GDP) and the total discounted costs (discount rate = 3%) of adaptation costs and residual expected flood damages over the full time horizon (2020–2150). Results are shown per country and for Europe as a whole under the SSP5-8.5 scenario.

Country	Coast length (km)	Protection (%)	Retreat (%)	Accommo-dation (%)	Protect and retreat (%)	Nothing (%)	Adaptation costs in 2020 (bil. US$)	Adaptation costs in 2020 (% of 2023 GDP)	Total adaptation costs (bil. US$)	Total residual flood damage (bil. US$)
Åland	3886	0.0	5.5	0.0	0.0	94.5	0.0	-	0.2	0.0
Albania	753	43.6	10.9	0.0	0.0	45.5	0.7	3.1	1.8	26.1
Belgium	319	96.5	0.6	0.0	2.2	0.7	3.4	0.5	11.6	2.1
Bulgaria	466	11.4	12.5	0.0	1.2	75.0	0.0	0.0	0.1	0.9
Croatia	5309	3.3	8.1	0.0	1.0	87.6	0.4	0.4	1.4	0.5
Cyprus	641	3.3	7.7	0.0	1.9	87.0	0.0	0.1	0.1	0.2
Denmark	6527	12.2	59.2	0.2	3.3	25.2	12.4	3.1	36.9	5.9
Estonia	2559	0.0	49.3	0.3	0.1	50.3	0.1	0.3	2.9	0.0
Faroe Islands	919	1.1	15.3	0.0	0.0	83.6	0.2	-	0.6	0.4
Finland	17922	3.0	28.4	0.0	2.9	65.7	6.4	2.1	16.6	2.8
France	8942	21.1	28.3	0.1	9.7	40.9	20.7	0.7	56.9	20.8
Germany	4952	46.5	33.3	0.1	4.3	15.8	30.7	0.7	92.3	32.1
Gibraltar	14	29.9	0.0	0.0	0.0	70.1	0.0	NaN	0.1	0.0
Greece	14267	7.4	7.3	0.0	0.8	84.5	0.2	0.1	2.9	7.3
Iceland	7971	0.7	35.7	0.4	0.8	62.3	5.3	17.0	6.0	25.2
Ireland	6630	3.9	38.5	0.0	1.9	55.7	5.2	0.9	22.5	1.5
Italy	8813	22.5	15.9	0.0	2.5	59.1	10.9	0.5	27.7	18.2
Latvia	684	16.8	42.4	0.0	16.4	24.4	2.6	6.1	5.1	1.3
Lithuania	356	42.1	45.4	0.0	0.0	12.5	0.4	0.5	3.3	1.1
Malta	190	18.5	6.1	0.0	0.0	75.4	0.0	0.1	0.1	0.9
Monaco	5	33.1	0.0	0.0	0.0	66.9	0.0	-	0.0	0.0
Montenegro	395	3.6	16.6	0.0	0.6	79.2	0.1	1.1	0.2	0.2
Netherlands	4523	75.2	13.8	0.1	1.1	9.8	58.6	5.2	137.5	912.9
Norway	52984	1.3	13.5	0.1	0.7	84.4	27.4	5.7	31.9	152.0
Poland	1507	38.4	32.2	1.4	12.7	15.3	10.2	1.3	18.3	7.0
Portugal	3273	2.6	28.6	0.1	10.5	58.2	1.4	0.5	3.9	0.3
Romania	1084	4.9	68.4	0.2	0.5	26.0	0.5	0.1	0.7	2.0
Slovenia	53	39.6	12.1	0.0	1.4	46.9	0.1	0.1	0.2	0.7
Spain	7648	14.4	11.9	0.1	6.6	67.1	3.2	0.2	11.7	3.6
Sweden	24115	2.0	20.4	0.2	1.5	75.9	8.2	1.4	13.1	13.9
Turkey	827	4.3	6.7	0.0	0.3	88.7	0.0	0.0	0.1	2.0
Ukraine	1732	0.0	23.2	0.0	0.1	76.7	0.1	0.1	0.1	0.1
United Kingdom	19867	9.7	24.1	0.1	3.9	62.1	43.3	1.3	134.3	45.1
Total	214891	8.7	21.1	0.1	2.4	67.6	253.0	1.1	640.9	1287.0

Costs and damages are given in 2024 values and expressed as a percentage of each country's GDP for 2023. Only the European coastlines of Russia and Turkey are reported.

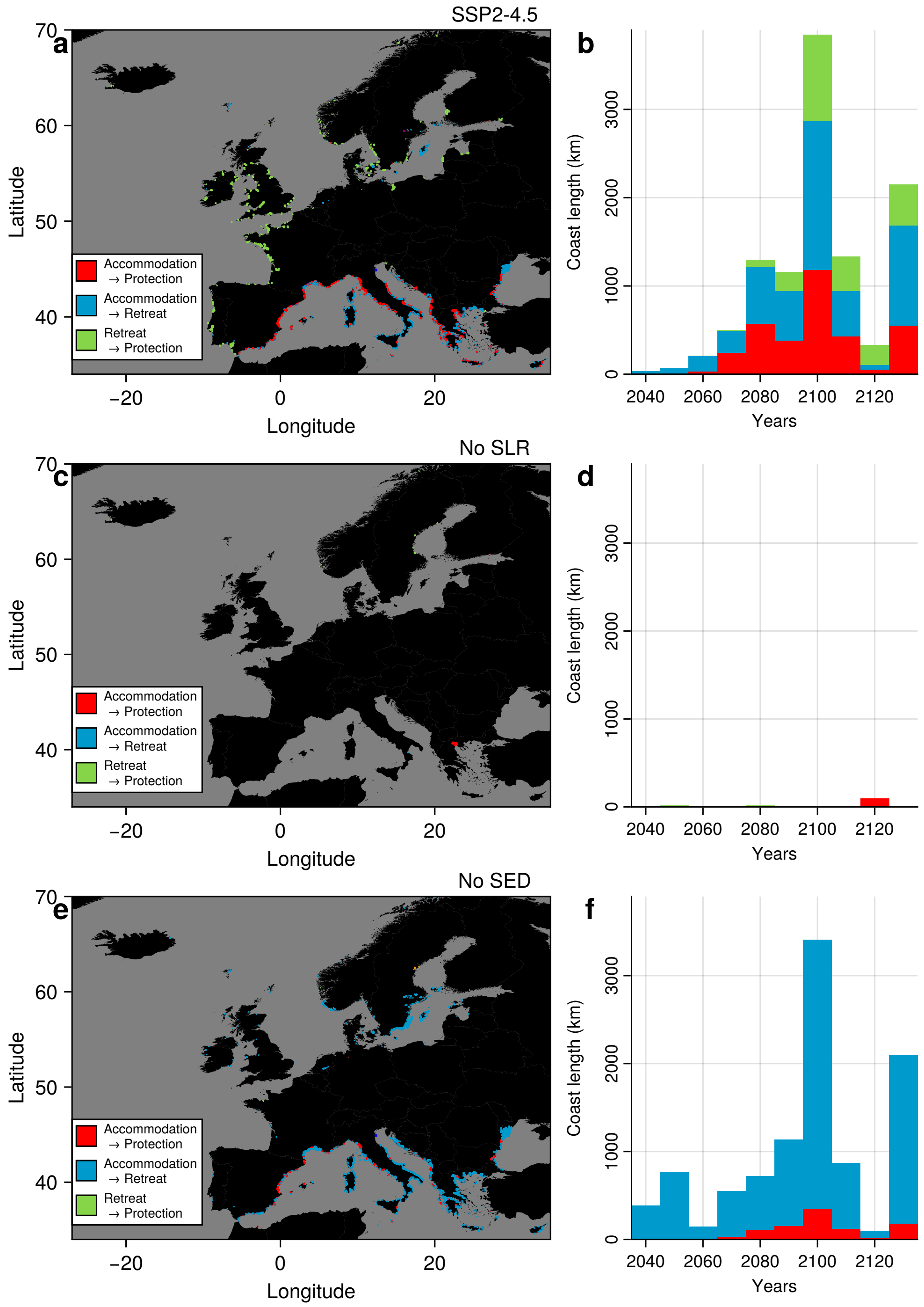

Fig. S1: **The geographic location and timing of adaptation tipping points excluding SLR and socioeconomic development (SED). a,c,e,** Maps showing the geographic location of floodplains where certain ATP occur under (**a**) SSP2-4.5, (**c**) excluding SLR and (**e**) excluding SED. **b,d,f,** Stacked bar charts illustrating the timing of ATP (x-axis) for the accumulated length of coastline (y-axis) for all floodplains under (**b**) SSP2-4.5, (**d**) excluding SLR and (**f**) excluding SED, using the same color codes as in (**a**), (**c**), and (**e**). Eurostat (GISCO), 2024. Licensed under CC BY 4.0: https://creativecommons.org/licenses/by/4.0/ © EuroGeographics for administrative boundaries in the basemaps.

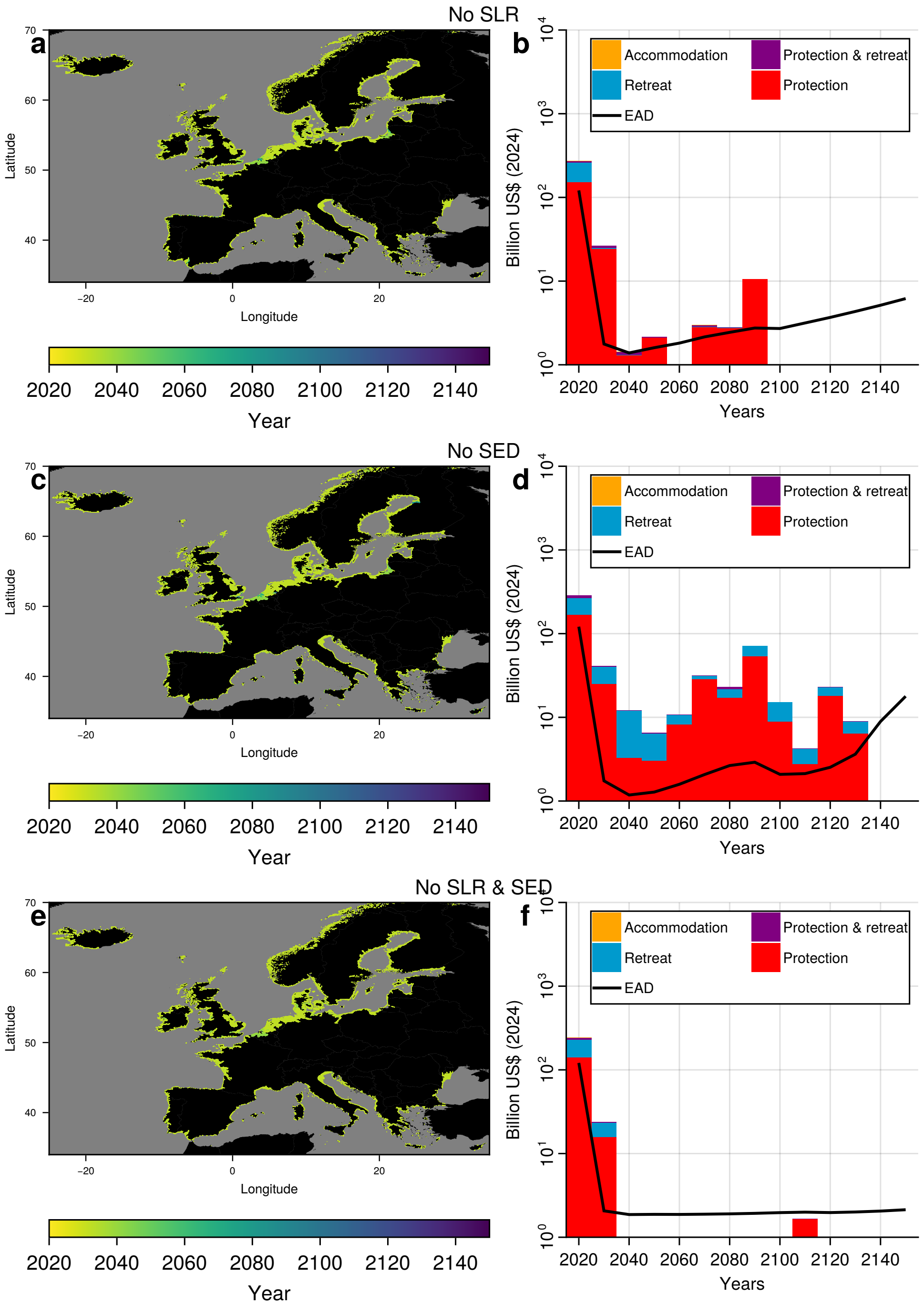

Fig. S2: **The timing of adaptation actions excluding SLR and socioeconomic development (SED). a,c,e,** Maps showing the year of implementation for the very first adaptation action for each floodplain (**a**) excluding SLR, (**c**) excluding SED and (**e**) excluding SLR and SED. **b,d,f,** Graphs showing adaptation investments for each adaptation option (stacked bar plots) and expected annual flood damage (EAD) (black line), both shown over time and aggregated across all floodplains, (**b**) excluding SLR, (**d**) excluding SED and (**f**) excluding SLR and SED. Eurostat (GISCO), 2024. Licensed under CC BY 4.0: https://creativecommons.org/licenses/by/4.0/ © EuroGeographics for administrative boundaries in the basemaps.

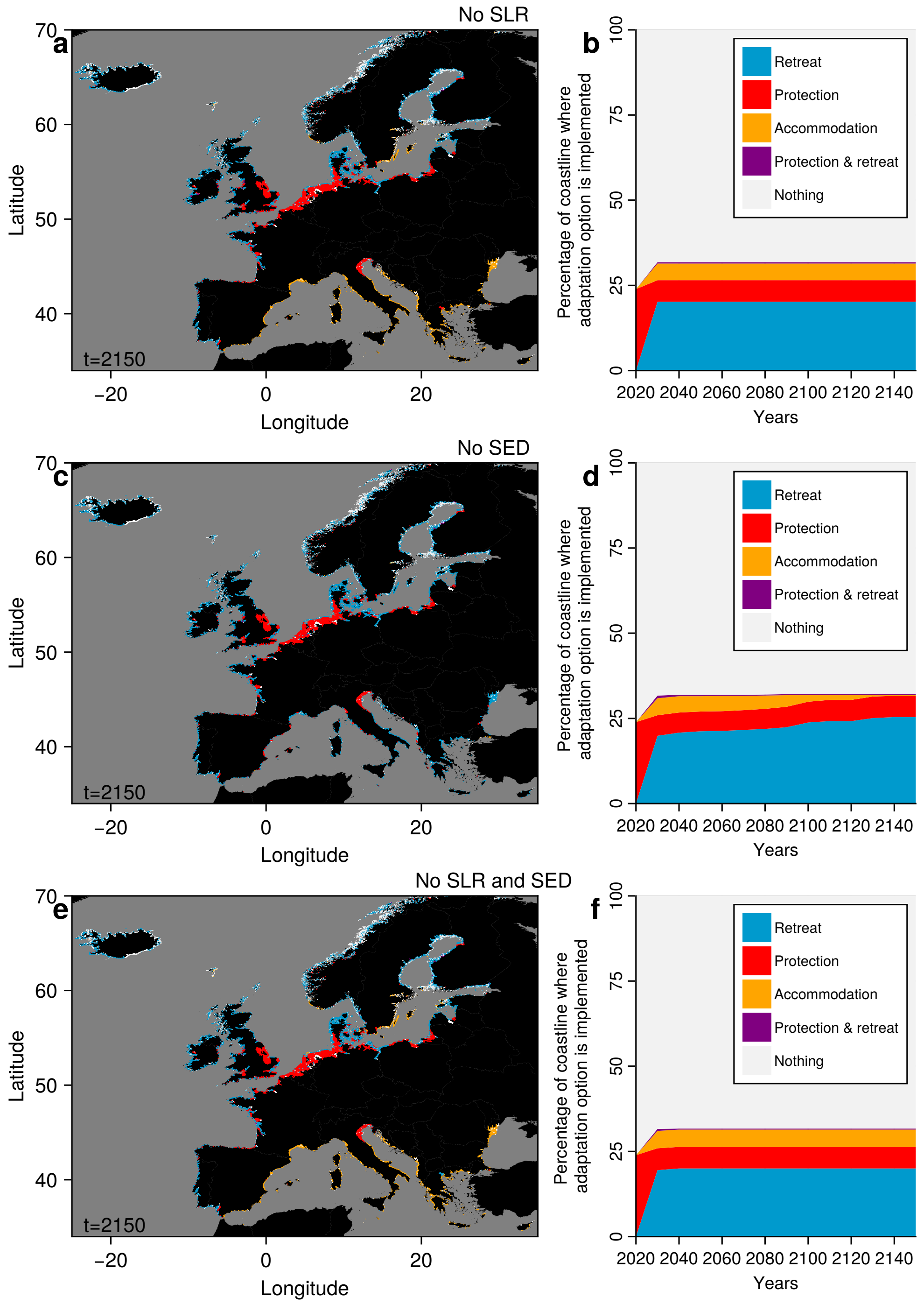

Fig. S3: **Implemented adaptation options across floodplains and over time excluding SLR and socioeconomic development (SED). a,c,e,** Maps showing the final adaptation option implemented per floodplain in 2150 (**a**) excluding SLR, (**c**) excluding SED and (**e**) excluding SLR and SED. **b,d,f,** Graphs showing the percentage of the entire coastline where each adaptation option is implemented over time under (**a**) excluding SLR, (**c**) excluding SED and (**e**) excluding SLR and SED. Eurostat (GISCO), 2024. Licensed under CC BY 4.0: https://creativecommons.org/licenses/by/4.0/ © EuroGeographics for administrative boundaries in the basemaps.

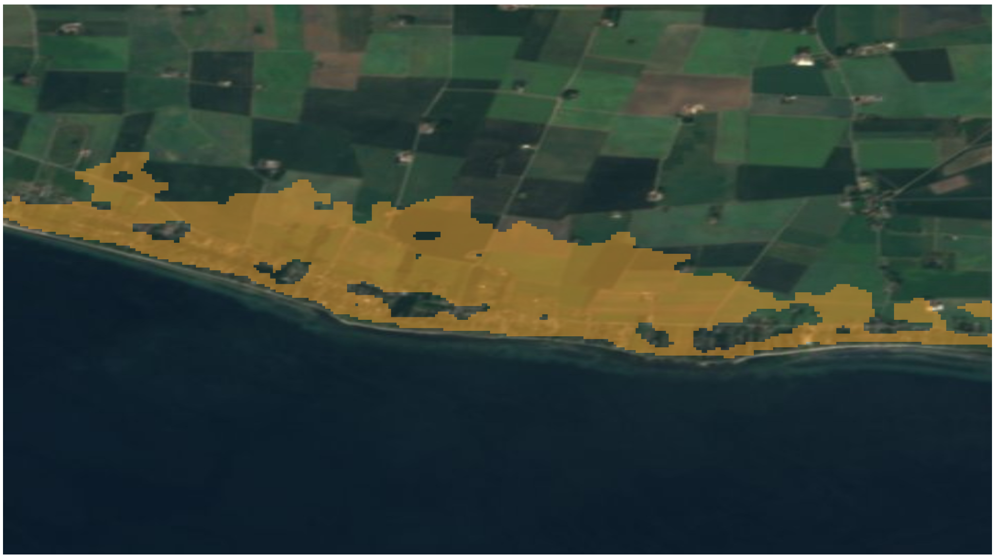

Fig. S4: **ATP from retreat to protection in Simremarken, Sweden, under SSP5-8.5.** The map illustrates the municipality of Simremarken and its surrounding areas, with the floodplain highlighted in transparent orange. Under SSP5-8.5, the economically optimal adaptation pathway involves a retreat of up to 1.4 meters in less developed areas by 2030, followed by the implementation of new protection for the remaining settlements in 2110 to protect the city. (Contains Copernicus Sentinel data (2016 & 2017)).
