# Supplementary material for: The economically optimal mix and timing of coastal adaptation in Europe to 2150
Source: Nat Commun. 2026 Jul 15;17:6249. doi: 10.1038/s41467-026-74042-8 (PMC13373216; doi:10.1038/s41467-026-74042-8)
Supplement: Supplementary file 2 — Transparent Peer Review file [file 41467_2026_74042_MOESM2_ESM.pdf]

nature portfolio

Peer Review File

The economically optimal mix and timing of coastal adaptation in Europe to 2150

Corresponding Author: Ms Vanessa Völz

This file contains all reviewer reports in order by version, followed by all author rebuttals in order by version.

Version 0:

Reviewer comments:

Reviewer #1

(Remarks to the Author)
Overall comments

This paper covers an important topic related to when and how to adapt and take an economic perspective on this. The authors build on the approaches of adaptation tipping points and adaptation pathways, and add an assessment of economic instead of traditional performance assessment. Moreover they add to the current literature on adaptation at continental scale, which is scarce in itself, but they add an exploration on the timing of adaptation tipping points and exploring pathways for coastal adaptation, with interesting findings on adaptation strategies from an economic perspective.

We have enjoyed reading the manuscript. The manuscript is well written, in particular the line of thought and order in which results are presented are very clear. The figures are also clear.

There are a few comments and unclarities that we think need to be addressed before publishing:
• The paper lacks a definition of what is considered economically optimal, and for who? What determines an ATP? I assume it is flood damage compared to costs? It would be good to highlight this in the main text. Similarly, how do you define 'economic under-adaptation'? (L32)
• Also relating to ATPs, you define them as 'moments requiring a switch between options, often due to economic limits' (L26 in abstract, but same definition used throughout). Although an economic definition of ATP has been used before in literature and is relevant, this is not the only way to define an ATP. In fact, in the literature it is mostly reduced risk that is a reason for an ATP. I would thus expect you to position the way you define an ATP with other definitions in the literature, for example by mentioning applications that relate to growing risks (and highlight that this is the most used definition), social acceptability, technical feasibility, etc. See for example https://wires.onlinelibrary.wiley.com/doi/full/10.1002/wcc.64 and how the concept has further developed since then. Similarly, please note how ATPs may be defined not only as 'moments in time when it is necessary to switch from one option to another (P2 L58), but also conditions under which this is the case.
• As recognized in the paper locally adaptation pathways can be different. This work could inform and give a first order assessment of what could be locally economically optimal. This could be added to P2, L106
• It is interesting to start with three major cities, to understand the process and outcomes of this study. It would be useful if you could introduce the three cities right at the beginning of the paragraph, including how they are different from each other (and thus why you picked these 3 specifically) and whether they are made up of multiple floodplains (and how the floodplains differ from each other, central floodplains vs floodplains with low-asset values), so that the text is more self-explanatory even without looking at Figure 1.
For one of these cities, London, an adaptive pathways plan with adaptation tipping points exists. Also, for the Netherlands an adaptive plan exists. A verification and discussion on a comparison with local adaptation plan could be beneficial.

• Figure 1 shows 3 different pathways. Does it mean it is possible to swich between these pathways. DO we understand well that for London, we see a pathway of protect until ~2030 then retreat. However, it seems unlogic and not economic optimal to do only protect for such short period. Or does this mean continue with protect to 2030 and then retreat? This is unclear.
• Overall, the spatial differences across Europe are very well described and clear, whereas the timing component could be better specified in text, especially in section 1.3 of the Results. See below for a couple of examples of where the text would benefit from more detail on this.
• If adaptation costs are much smaller than residual costs, would more adaptation be possibly economically optimal? Or is

more adaptation then more costly?
• Are transfer costs considered? E.g. switching pathways could add to costs because of path-dependencies. I don't expect this to be included as this maybe largely dependent on local conditions, but it would be good to mention this. We have explored this in this paper on economic evaluation of pathways that influences the ranking of pathways https://doi.org/10.1007/s10584-019-02409-6. What pathway is more economically optimal?
• The Discussion compares outcomes with other studies, but lacks a discussion on the limitations. What are the limitations of your approach? E.g. what you define as economically optimal, the adaptation measures you consider, the spatial scale of your analysis, (lack of) consideration of different socioeconomic scenarios (e.g. an increase in development of low-density coastal areas). You also did not look at advance/seaward adaptation options.

Detailed comments
• Abstract contains a typo: adaption   adaptation. I saw it also in the introduction
• P2, L35: I assume 30 years, is average, as also mentioned elsewhere in the manuscript.
• P2, L47: I would recommend not use the term 'threshold' here, as this is often used interchangeably with ATP. You may for example rephrase as 'Exceeding/Reaching higher extents/values of SLR...'
• P3, L77: here you mean the previous global assessments? As this has been done at local scale.
• P3, L84: some global assessments on timing of adaptation tipping points/thresholds have been done, although linked to economic: https://www.nature.com/articles/s41558-023-01616-5 and https://www.sciencedirect.com/science/article/pii/S221209632100084X
• P3, L100: '...addressing the limitations of previous single-stage cost-benefit analyses, which overestimate the costs due to constant adaptation options over time': Please further explain why single-stage costs benefit analyses will always overestimate the cost of adaptation, a reference would also be useful here.
• P4, L120: 'However, even in proximity to major urban cities such as London and Venice...': Using the word 'even' does not work here because this is the first example of this you are presenting in your results (and this is the first result section overall), so the reader does not yet have specific expectations on this.
• P4, L134: 'In all adaptation pathways of the three cities...' I find this sentence unclear; how many adaptation pathways are there for each city? The second half of this sentence is also somewhat complex and could be made clearer.
• P4, L138: Please specify the timing of the first timestep. You explained this before but is useful to repeat here to enhance clarity.
• P4, L143: The sentence starting with 'Under the assumption of currently higher protection levels...' is unclear.
• P4, L53: 'Retreat is the economically optimal response for 26% of the total length of Europe's coastline' When is this the case? For which timestep(s)? Do you mean retreat as a first option?
• Similarly, in P5, L164 '..protection is implemented along 97%, 68% and 33% of their total coastline, respectively' By when is this the case? By the end of your projections (2120)? Or at any point in the next 100 years?
• If space allows, the point on P5, L183-184 would be interesting for the abstract
• P6, Table 1 capture: 'The proportion of the coastline for which different adaptation options are implemented in 2120' Do you mean implemented in 2150, or (at any point) by 2150?
• Section 1.5 of the results would very much benefit from a graph showcasing the development of AED (either averaged for Europe, or of a relevant specific floodplain/country). This would make the points you make in text even clearer. If there is no space for this in the main text, an addition in annexes would also be appreciated.
• P6, L223: 'However, EAD rises again towards the end of the time horizon, as adaptation investments to maintain protection levels under rising sea levels cease after 2130'. I do not understand what you are trying to say here.
• P5, L236: There is a typo in the word 'extent'

Marjolijn Haasnoot,
Valeria Di Fant
Delft, 2025

(Remarks on code availability)

Reviewer #2

(Remarks to the Author)
I co-reviewed this manuscript with one of the reviewers who provided the listed reports. This is part of the Nature Communications initiative to facilitate training in peer review and to provide appropriate recognition for Early Career Researchers who co-review manuscripts.

(Remarks on code availability)

Reviewer #3

(Remarks to the Author)
Thank you for the opportunity to review this manuscript, "The economically optimal mix and timing of coastal adaptation in Europe to 2150". The study advances research on coastal adaptation by providing large-scale quantitative evidence on the most economically optimal coastal adaptation pathways for coastal Europe. Unlike previous European coastal flood risk assessments, this work considers multiple adaptation strategies (i.e., protection, retreat, and accommodation), which

enables the authors to identify points where the economically optimal strategy shifts (e.g., from accommodation to retreat). Notably, because the study includes multiple strategies and allows strategies to shift over time, the study projects a lower estimation of expected annual flood damages than previous efforts. The study focuses on a single hazard (coastal flooding) but does so at a higher spatial resolution than past efforts and considers 41,327 coastal floodplains.

Overall, this work advances the methods for adaptation decision-making science, and we recommend for publication with a few revisions to clarify interpretation of the results. We (co-reviewers) share a few opportunities for the authors to improve this manuscript below. The first group are edits we feel are important for the paper to be published, primarily focused on clarifying methods and a few revisions to figure captions. The second group are suggestions that we believe would strengthen the manuscript.

Concerns Needing Review

We had several questions about the methods and how choices about costs and limits affect interpretation of the results.

1. Authors describe accommodation as a binary variable indicating whether all homes in a 1-meter floodplain have been floodproofed to 1 meter in height. This appears to mean that accommodation, unlike retreat and protection, does not have a height variable higher than 1 meter. If this is incorrect, we ask the authors to clarify. If correct, the decision to limit accommodation to 1m high could be driving the study's findings that many areas in Europe will eventually shift from accommodation to retreat or protection (i.e., if accommodation were possible to a higher height, would this tipping point still occur?). We are not recommending additional analyses but that the authors discuss how this accommodation height affects the results and why 1m was selected as the limit. In the US, there are many structures that have undergone accommodation beyond 1 meter, so accommodation beyond this level is possible from engineering and economic perspectives.

2. The authors estimate retreat costs at three times the local GDP per capita per migrant. We find that estimation to be low, as it is less than the median home value in many European countries. Retreat costs vary depending on the type of retreat (e.g., autonomous, government acquisitions, community relocations) but many government-invested retreat programs compensate by home value, so a cost below median home value seems low. The authors note that they conducted a sensitivity analysis using retreat costs up to 6x GDP per capita. How did that sensitivity analysis affect the results? At 6x GDP, is an early investment in retreat still considered economically optimal?

3. One significant result that currently feels buried is that it is economically optimal to retreat from 99% of the retreated coast (26% total) in the first time step. (Although, as per our previous point, is this still true if retreat costs double?) How did the authors consider the short-term economic benefits gained from coastal development? That is, what are the lost economic opportunities associated with retreat at an early phase? Whether those losses are none or are outweighed by the gains in avoided damage from flooding, either way the result is surprising and merits highlighting. If the missed opportunity costs were not considered, that is a limitation worth noting in the discussion or methods.

4. A few minor suggestions for figure captions:
o There is some confusing text in the caption of Figure 5, including "shown in blue (green)".
o Figure 1: It would be useful to describe in the caption that "height of retreat" means the height above sea level below which infrastructure is removed. This is stated in the methods section but is needed here for a reader to understand the figure.
o Figure 2: the caption says "both" but there are three graphs, not two. Is the EAD line truly at $0 between 2030 and 2100? (From the text, it appears not, but the graph makes it look like $0.) If so, that is remarkable and a result worth highlighting. If not, it would be worth clarifying the actual scale of the EAD and that the line appears to reach 0 due to scaling on the graph. The caption also needs to explain the "X EAD Vousdoukas" to enable a reader to interpret the graph alone.

Minor Notes / Suggestions

1. We recommend revising the abstract to focus less on adaptation tipping points (ATP) and more on other surprising results. Although including ATP in the analysis is a methodological advance, the paper has fewer surprising findings about ATP. Reframing the abstract and introduction to highlight the most surprising and important results of the paper will help this work be read by the wider audience it deserves.

Findings we found particularly interesting include:
a. The results show that retreat is economically optimal for one-fourth of Europe's coastline and that the adaptation costs for protection exceed those for retreat. This is a surprising finding and useful information for adaptation decision-makers, given Europe's history of choosing protection over other options.

b. The analysis shows that for 99% of coastlines needing adaptation, the optimal timing for initial actions is immediate (in the first time step), and the initial investments are significant (Figure 2). The idea that widespread action is needed urgently is not surprising, but the extreme nature of the results in this study are surprising and worth highlighting.
i. This also raises a question: Did the authors place any limits on adaptation investments (e.g., a limit that nations do not invest more than 20% of their GDP in a given decade)? Or a sensitivity analysis to see what happens if nations do not invest in adaptation in the first time step? Additional analyses are not required, but we were surprised by the extreme costs in the first step (Figure 2) and wondered if that level of adaptation investment is economically or politically possible. If not, the analysis provides an interesting hypothetical but not an applicable guide.

c. Table 1: The final column appears to compare the costs of adaptation and flood damages (totaled over 130 years) to

national GDP in 2023. This temporal mismatch makes it unclear how to use that final column. What does it tell us that adaptation costs and flood damages combined over the coming century will cost 8.2% of Europe's GDP? Would it not be more relevant to compare the adaptation costs in the first time step to 2023 GDP? Or to compare the coming century of adaptation and flood damages to the coming century of GDP? Given the extreme adaptation costs that are optimal in the first time step, it would be fascinating to see a comparison of the recommended adaptation investments in the first time step for each country relative to that nation's GDP. That would provide a very useful insight into the feasibility of the recommended investments.

d. The phasing of retreat to protection was surprising. It makes sense, especially where retreat to higher ground results in denser infrastructure that then merits protection. However, this phasing is rarely discussed in the adaptation literature and is a notable contribution to discussions around adaptation pathways.
i. In Lines 292-299, the authors discuss retreat as a "last resort" when no ATP are reached after retreat. While we appreciate the author's point about protection at higher elevations after (or in coordination with) retreat, we caution on the "last resort" language because of the spatial issue. Namely, protection at higher elevation after retreat has occurred at lower elevation still involved retreat as a "last resort" for the people living at those lower elevations. A controversial example from the United States is the relocation of a tribal community on Isle de Jean Charles. A system of protected levees was built inland of the island (and at higher elevation), leaving the islanders with few options other than retreat. The fact that the region combined protection, accommodation, and retreat does not make retreat less of a last resort for this Tribe. We recommend a minor edit to change the last resort language to phrasing that acknowledges the combination of strategies.

2. The authors say that 68% of the total length of Europe's coastline will not require adaptation because it is not exposed to flood risks and SLR. Does the analysis also consider coastal erosion rates (we see a note about vertical land movement in section 3.2.2 but no discussion of erosion)? Or interactions with inland and riverine flooding? (In London and much of the Netherlands, for example, the interaction between coastal and riverine flooding is a major consideration in adaptation planning.) Our suggestion is not to include erosion or riverine flooding but to provide some discussion on how the authors think inclusion of additional hazards might change the results of their analysis.

3. Why did the authors choose London, Venice, and Thessaloniki as examples? Venice and London, in particular, are known for being unusual in terms of their exposure (e.g., London being affected by coastal and riverine flooding and being a somewhat non-traditional "coastal" city; and Venice being a heritage icon). This may lead readers (as it did to us) to fixate on the particulars of those cities, whereas a less iconic city might help illustrate the authors' analysis and the value of the findings more clearly.

4. In the introduction, the authors note that ATP is driven by economic and social factors. Line 200-202 notes that ATP "can be completely attributed to SLR" (within the model) because it does not occur in a counterfactual situation. Both of these statements may be true, but it would be interesting to see the authors discuss why they think SLR drives ATP: why projected population and GDP growth rates, for example, were insufficient to drive ATP.

We hope that this review is useful to the authors, and we look forward to the publication of this manuscript.

(Remarks on code availability)

Reviewer #4

(Remarks to the Author)
I co-reviewed this manuscript with one of the reviewers who provided the listed reports. This is part of the Nature Communications initiative to facilitate training in peer review and to provide appropriate recognition for Early Career Researchers who co-review manuscripts.

(Remarks on code availability)

Version 1:

Reviewer comments:

Reviewer #1

(Remarks to the Author)
Our comments have been well addressed and congratulate the authors with this important contribution to the literature on coastal adaptation. For example, the introduction to the example cities, why they were chosen and how they are different from each other is now a lot clearer. Also, we are happy to see the added definition of economic ATP, under-adaptation and economically optimal adaptation.

(Remarks on code availability)

Reviewer #2

(Remarks to the Author)
I co-reviewed this manuscript with one of the reviewers who provided the listed reports. This is part of the Nature Communications initiative to facilitate training in peer review and to provide appropriate recognition for Early Career Researchers who co-review manuscripts.

(Remarks on code availability)

Reviewer #3

(Remarks to the Author)
Thank you for the thorough and thoughtful revisions. The authors have addressed all of our concerns, and the revised manuscript reads more clearly, with stronger articulation of its implications. I have no remaining substantive concerns and look forward to seeing the paper published.

(Remarks on code availability)
This type of modeling is not our methodological forte (nor do we code in Julia), so we visited the Zenodo site and can confirm that the data, code, and results are available but we did not attempt to reproduce any portion of the analysis.

Reviewer #4

(Remarks to the Author)
I co-reviewed this manuscript with one of the reviewers who provided the listed reports. This is part of the Nature Communications initiative to facilitate training in peer review and to provide appropriate recognition for Early Career Researchers who co-review manuscripts.

(Remarks on code availability)
I co-reviewed this manuscript with one of the reviewers who provided the listed reports. This is part of the Nature Communications initiative to facilitate training in peer review and to provide appropriate recognition for Early Career Researchers who co-review manuscripts.

Open Access This Peer Review File is licensed under a Creative Commons Attribution 4.0 International License, which permits use, sharing, adaptation, distribution and reproduction in any medium or format, as long as you give appropriate credit to the original author(s) and the source, provide a link to the Creative Commons license, and indicate if changes were made.
In cases where reviewers are anonymous, credit should be given to 'Anonymous Referee' and the source.
The images or other third party material in this Peer Review File are included in the article’s Creative Commons license, unless indicated otherwise in a credit line to the material. If material is not included in the article’s Creative Commons license and your intended use is not permitted by statutory regulation or exceeds the permitted use, you will need to obtain permission directly from the copyright holder.
To view a copy of this license, visit https://creativecommons.org/licenses/by/4.0/

Response to the reviewers' comments

Manuscript Number: NCOMMS-25-30749-T
Title: The economically optimal mix and timing of coastal adaptation in Europe to 2150

Responses to review comments are provided in blue italics font below the review comments.

Reviewer #1 (Remarks to the Author):

Overall comments

This paper covers an important topic related to when and how to adapt and take an economic perspective on this. The authors build on the approaches of adaptation tipping points and adaptation pathways, and add an assessment of economic instead of traditional performance assessment. Moreover they add to the current literature on adaptation at continental scale, which is scarce in itself, but they add an exploration on the timing of adaptation tipping points and exploring pathways for coastal adaptation, with interesting findings on adaptation strategies from an economic perspective.

We have enjoyed reading the manuscript. The manuscript is well written, in particular the line of thought and order in which results are presented are very clear. The figures are also clear.

We thank reviewer #1 and #2 for this detailed review and are pleased to hear that they think this manuscript is adding value to current literature on adaptation at continental scale and that results are clearly communicated. We address each question and comment of reviewer #1 and #2 below. We would like to thank reviewer #1 and reviewer #2 for helping us to improve our manuscript with their comments.

There are a few comments and unclarities that we think need to be addressed before publishing:

- The paper lacks a definition of what is considered economically optimal, and for who? What determines an ATP? I assume it is flood damage compared to costs? It would be good to highlight this in the main text. Similarly, how do you define 'economic under-adaptation'? (L32)

We agree with the reviewers that a clearer definition of "economically optimal" is beneficial, and we have revised the Introduction accordingly:

"For the first time, we offer large-scale quantitative evidence on adaptation pathways for all of coastal Europe, thereby identifying economically optimal coastal adaptation pathways, including economic ATP, for each individual floodplain. Here, economically optimal refers to the adaptation option that minimizes the combined costs of flood damages and adaptation actions from a public economic perspective."

We also clarify economic under-adaptation in line 32:

"For 99% of floodplains requiring adaptation, the optimal timing for initial actions is immediate due to current economic under-adaptation, a condition in which the costs of additional adaptation are outweighed by the reduction in expected flood damages."

For a definition of ATPs, please see our response to the next comment.

- Also relating to ATPs, you define them as 'moments requiring a switch between options, often due to economic limits' (L26 in abstract, but same definition used throughout). Although an economic definition of ATP has been used before in literature and is relevant, this is not the only way to define an ATP. In fact, in the literature it is mostly reduced risk that is a reason for an ATP. I would thus expect you to position the way you define an ATP with other definitions in the literature, for example by mentioning applications that relate to growing risks (and highlight that this is the most used definition), social acceptability, technical feasibility, etc. See for example https://wires.onlinelibrary.wiley.com/doi/full/10.1002/wcc.64 and how the concept has further developed since then. Similarly, please note how ATPs may be defined not only as 'moments in time when it is necessary to switch from one option to another (P2 L58), but also conditions under which this is the case.

Thank you for this important comment on the broader definitions of ATPs in the literature. We have addressed this by expanding our Introduction with the following addition:

“While this study emphasizes economic ATPs, the original concept was defined more broadly as situations where the magnitude of change prevents an option from meeting its objectives (Kwadijk et al., 2010). In the literature, ATPs are most often associated with risk thresholds, but they may also result from physical, ecological, technical, societal, political, or - as explored here - economic limits (Haasnoot et al., 2024).”

We have also refined our working definition of ATPs:

“Crucially, such pathways potentially entail adaptation tipping points (ATP), moments or conditions in time when it is necessary to switch from one option to another, such as, e.g., from protection to retreat (Kwadijk et al., 2010).”

- As recognized in the paper locally adaptation pathways can be different. This work could inform and give a first order assessment of what could be locally economically optimal. This could be added to P2, L106

We thank the reviewers for this valuable suggestion and have adjusted the corresponding sentence accordingly:

“While these insights into the economically optimal sequencing and timing of adaptation options provides a crucial source of information for European and global level policy decisions (Bisaro et al. 2021), and offer a first-order assessment of what may be locally economically optimal, we note that economic considerations can and should not be the only type of information used in coastal decisions.”

- It is interesting to start with three major cities, to understand the process and outcomes of this study. It would be useful if you could introduce the three cities right at the beginning of the paragraph, including how they are different from each other (and thus why you picked these 3 specifically) and whether they are made up of multiple floodplains (and how the floodplains differ from each other, central floodplains vs floodplains with low-asset values), so that the text is more self-explanatory even

without looking at Figure 1. For one of these cities, London, an adaptive pathways plan with adaptation tipping points exists. Also, for the Netherlands an adaptive plan exists. A verification and discussion on a comparison with local adaptation plan could be beneficial.

We thank the reviewer for this constructive suggestion. We have revised the first and second paragraphs of our Results section accordingly:

"To illustrate the general nature of our approach, we first present results for three major European cities under SSP2-4.5, representing different geographic and socio-economic characteristics in terms of extreme sea levels, population density, and current protection levels. The cities are: London, a mega-city with very high coastal flood protection; Venice, a large city with medium protection levels and UNESCO World Heritage status; and Thessaloniki, a large city without any large coastal flood protection today. In our model, each city and its surrounding regions include multiple floodplains, spanning densely populated urban areas as well as rural zones with low asset values. We focus on two representative floodplains per city (Figure 1)."

"London has an adaptive plan for coastal flooding over the next century (Environment Agency). While the detailed proposals cannot be compared with the results of this model, qualitatively there is good agreement with this analysis showing incremental upgrades of protection levels at a rate depending on the rate of sea-level rise, leading to comparable overall outcomes."

- Figure 1 shows 3 different pathways. Does it mean it is possible to swich between these pathways. DO we understand well that for London, we see a pathway of protect until ~2030 then retreat. However, it seems unlogic and not economic optimal to do only protect for such short period. Or does this mean continue with protect to 2030 and then retreat? This is unclear.

We thank the reviewers for highlighting that the interpretation of our adaptation pathway plots was unclear. The reviewers' understanding is correct: for one relatively undeveloped floodplain in London downstream of the core of the city, the pathway shows continued protection until 2030, followed by a switch to retreat. This represents the continuation of protection that is already in place in the respective floodplain, visualized by the protection line starting above 0 in the first year of the model, which is maintained until 2030 and then replaced with retreat. To clarify this in the manuscript, we added the following explanation:

“However, even in proximity to major urban centres such as London and Venice, retreat is an economically optimal option for floodplains containing low asset values such as those used for agriculture, nature reserves or green space. The largest floodplains of this kind identified for retreat in our three examples are located east of London on the outer Thames Estuary marshes and south of Venice in the Po Estuary, spanning coastal lengths of 66 km and 68 km, respectively. In these areas, current protection is maintained until 2030, after which retreat is implemented (Figure 1).”

- Overall, the spatial differences across Europe are very well described and clear, whereas the timing component could be better specified in text, especially in section 1.3 of the Results. See below for a couple of examples of where the text would benefit from more detail on this.

We thank the reviewers for their careful review and agree that the timing of economically optimal options was not always clearly stated in Section 1.3. We have now added the respective years in several parts of this section to provide a clearer temporal context.

- If adaptation costs are much smaller than residual costs, would more adaptation be possibly economically optimal? Or is more adaptation then more costly?

We thank the reviewers for this insightful comment. In our model, each floodplain has a maximum protection and retreat height defined as the 1-in-10,000-year extreme event height plus projected sea-level rise by 2150. For example, in London under medium climate change, this corresponds to a maximum protection height of 9.6 meters, whereas the economically optimal pathway reaches only 8 meters between 2080 and 2150 (Figure 1). This demonstrates that higher adaptation levels are considered in the model but would exceed the economically optimal level and not reduce overall costs.

The fact that only 32% of the net present value of overall costs comes from adaptation investments (Table 1) is largely due to the early years of the model, which are weighted more heavily through discounting. In the first decade (2020–2030), expected annual damages (EAD) of 121 billion USD accumulate to 1,210 billion USD over ten years, far exceeding the one-time adaptation investments of 246 billion USD made in this time step (Figure 2; now with an updated logarithmic y-scale showing EAD). Over the remainder of the time horizon, adaptation costs surpass residual damages, even when multiplying EAD by ten-year time steps, confirming that additional adaptation beyond the economically optimal level would not reduce overall costs.

We have added the following explanation in Section 1.5 to the manuscript to clarify this point:

"EAD start at US$ 121 billion in 2020 but decline significantly to US$ 0.8 billion in 2030 after initial adaptation investments are made in 2020, reflecting that Europe is substantially under-protected in the base year (Figure 2). Over the first decade (2020–2030), EAD accumulate to US$ 1,210 billion (5.4% of Europe's GDP in 2023), far exceeding the one-time adaptation investments of US$ 242 billion in 2020 (1.1% of Europe's GDP in 2023). After this initial adjustment, adaptation needs fall substantially and over much of the subsequent time horizon adaptation costs surpass residual damages, even when EAD is considered over full ten-year time steps (Figure 2)."

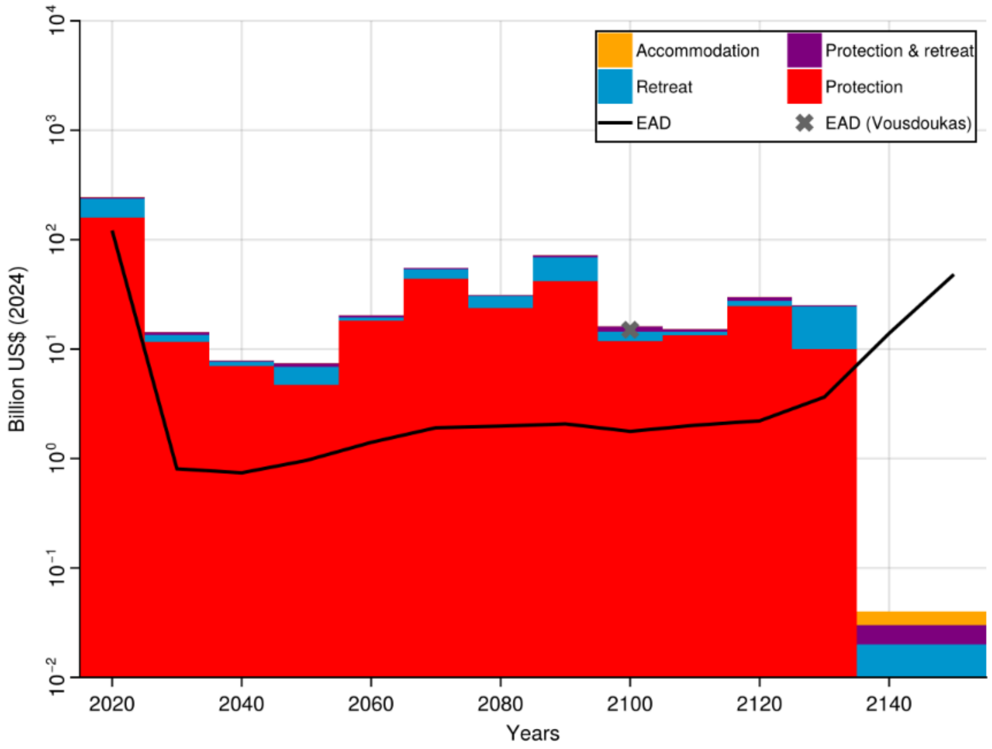

Adaptation investment and EAD in Figure 2 for SSP2-4.5.

- Are transfer costs considered? E.g. switching pathways could add to costs because of path-dependencies. I don't expect this to be included as this maybe largely dependent on local conditions, but it would be good to mention this. We have explored this in this paper on economic evaluation of pathways that influences the ranking of pathways https://doi.org/10.1007/s10584-019-02409-6. What pathway is more economically optimal?

We thank the reviewers for raising transfer costs. In our model, adaptation costs depend on the options already implemented at a given point in time. For example, the cost of upgrading dike height accounts for the current dike level, and a fixed-cost component makes multiple small upgrades more expensive than one large one. However, spatial constraints on dike construction or upgrading are not considered, which could substantially increase transfer costs. Retreat costs are calculated based on settlements that remain after earlier retreat actions below lower elevation thresholds. Thus, only the remaining settlements are evaluated for retreat costs. Importantly, our model does not account for transfer costs related to removing existing adaptation options, for instance, dismantling dikes when switching from protection to retreat. To clarify this, we have added the following explanation to our Methods section 3.1.2:

"We note that the model does not include spatial constraints on dike construction or upgrading, nor the costs of breaching or possibly dismantling dikes when shifting from protection to retreat. Both factors could substantially increase adaptation costs, especially when switching between options, as demonstrated in a recent study (Haasnoot et al.,2020)."

- The Discussion compares outcomes with other studies, but lacks a discussion on the limitations. What are the limitations of your approach? E.g. what you define as economically optimal, the adaptation measures you consider, the spatial scale of your analysis, (lack of) consideration of different socioeconomic scenarios (e.g. an increase in development of low-density coastal areas). You also did not look at advance/seaward adaptation options.

We agree with the reviewers that the previous version of our manuscript lacked a discussion of its limitations, and we thank them for drawing our attention to this important point. We have therefore added the following paragraph to the Discussion section:

"Our study has several limitations. The range of adaptation options considered is limited; for example, accommodation for higher sea levels or advance (land claim or raising) are not included, which could delay adaptation tipping points or enable new pathways. Socio-economic scenarios are represented only at broad scales, potentially overlooking local dynamics, such

as population growth in low-density coastal areas, which could increase retreat costs and residual flood damages. We focus solely on coastal flooding, whereas other related hazards, such as coastal erosion, heavy precipitation, and riverine flooding, are not considered. These hazards could require additional adaptation investments; for instance, global dike investments for riverine flooding could increase our adaptation costs in the first time step by roughly 36% (Ward et al, 2017). Other hazards may also interact with the adaptation options considered here - for example, coastal protection could exacerbate erosion or impede drainage during heavy precipitation. In our model, retreat is implemented in the first time step for 99% of floodplains. While this approach may prevent new coastal developments that could lock floodplains into a protection pathway, potential coastal development opportunities, such as ports, are not represented in the model. Finally, our analysis focuses exclusively on economic optimality, neglecting ecological, technical, societal, legal, and political dimensions of adaptation."

<u>Detailed comments</u>

- Abstract contains a typo: adaption adaptation. I saw it also in the introduction

Corrected.

- P2, L35: I assume 30 years, is average, as also mentioned elsewhere in the manuscript.

Yes, we added this.

- P2, L47: I would recommend not use the term ‘threshold’ here, as this is often used interchangeably with ATP. You may for example rephrase as ‘Exceeding/Reaching higher extents/values of SLR...’

Thank you for pointing out this potential confusion, we took up your suggestion.

- P3, L77: here you mean the previous global assessments? As this has been done at local scale.

Yes, we mean previous global assessments and highlighted this clearer.

- P3, L84: some global assessments on timing of adaptation tipping points/thresholds have been done, although linked to economic: https://www.nature.com/articles/s41558-023-01616-5 and https://www.sciencedirect.com/science/article/pii/S221209632100084X

Thank you for pointing us to these two references, we took them up with the following sentence:

"Moreover, non-economic global assessments consider adaptation tipping points and highlight the need for timely adaptation from a risk perspective in the coming decades (Hermans et al., 2023, Haasnoot et al., 2021)."

- P3, L100: '...addressing the limitations of previous single-stage cost-benefit analyses, which overestimate the costs due to constant adaptation options over time': Please further explain why single-stage costs benefit analyses will always overestimate the cost of adaptation, a reference would also be useful here.

Mathematically, solutions from single-stage cost-benefit analyses will always provide an upper bound for the costs obtained in multi-stage cost-benefit analyses, because the solution space of the latter is larger and always includes the optimal solution of the former. In other words, single-stage approaches cannot change adaptation actions over time, which can lead to both over- and under-adaptation as conditions, such as sea-level rise, change. We thank the reviewers for pointing out that references were missing for this statement. We now provide three references that quantify the overestimation of costs in single-stage approaches in local case studies and explain how over- and under-adaptation contribute to this:

"Our results provide an updated and more realistic estimation of costs for coastal adaptation and residual expected flood damages in Europe. This addresses limitations of previous single-stage cost–benefit analyses, which tend to overestimate costs by assuming constant adaptation options despite rising sea levels, thereby leading to both over- and under-adaptation over time (Truong et al, 2024; Skerker et al., 2023, Völz et al., 2024)."

- P4, L120: 'However, even in proximity to major urban cities such as London and Venice...': Using the word 'even' does not work here because this is the first example of this you are presenting in your results (and this is the first result section overall), so the reader does not yet have specific expectations on this.

Thank you, we deleted this.

- P4, L134: ‘In all adaptation pathways of the three cities...’ I find this sentence unclear; how many adaptation pathways are there for each city? The second half of this sentence is also somewhat complex and could be made clearer.

Thank you for pointing out that this sentence was unclear. We have revised it as follows:

“In all six adaptation pathways across the two representative floodplains per city (Figure 1), the very first adaptation actions are implemented in 2030. These actions include upgrading protection heights in densely populated floodplains (London and Venice), switching from protection to retreat in rural floodplains (near London and Venice), and implementing accommodation in floodplains in Thessaloniki.”

- P4, L138: Please specify the timing of the first timestep. You explained this before but is useful to repeat here to enhance clarity.

We agree and added the year.

- P4, L143: The sentence starting with ‘Under the assumption of currently higher protection levels...’ is unclear.

Thank you for pointing this out. We have clarified the sentence as follows:

“Assuming current protection levels are 20% higher in a sensitivity analysis, 8% of the European coastline requiring adaptation could delay its first adaptation actions until 2050 or later, compared with only 3% under the baseline scenario (Figure 5).”

- P4, L53: ‘Retreat is the economically optimal response for 26% of the total length of Europe's coastline’ When is this the case? For which timestep(s)? Do you mean retreat as a first option?

Thank you for pointing this out. We have clarified the sentence to include the relevant year 2150.

- Similarly, in P5, L164 ‘..protection is implemented along 97%, 68% and 33% of their total coastline, respectively’ By when is this the case? By the end of your projections (2120)? Or at any point in the next 100 years?

We also added the year 2150 there.

- If space allows, the point on P5, L183-184 would be interesting for the abstract

We thank the reviewers for suggesting that this point could be included in the abstract. We agree that it is interesting; however, the abstract has already reached the 150-word limit, which unfortunately prevents us from adding further findings on adaptation tipping points.

- P6, Table 1 capture: 'The proportion of the coastline for which different adaptation options are implemented in 2120' Do you mean implemented in 2150, or (at any point) by 2150?

We confirm that Table 1 refers to adaptation options implemented in 2150, and we have double-checked all tables to ensure consistency.

- Section 1.5 of the results would very much benefit from a graph showcasing the development of AED (either averaged for Europe, or of a relevant specific floodplain/country). This would make the points you make in text even clearer. If there is no space for this in the main text, an addition in annexes would also be appreciated.

We thank the reviewers for highlighting that a visualization of EAD strengthens Section 1.5. In response, we updated Figure 2 (see below) to include a logarithmic y-axis, illustrating both adaptation investments and EAD over time. This addition makes the temporal dynamics clearer and directly supports the findings discussed in the text.

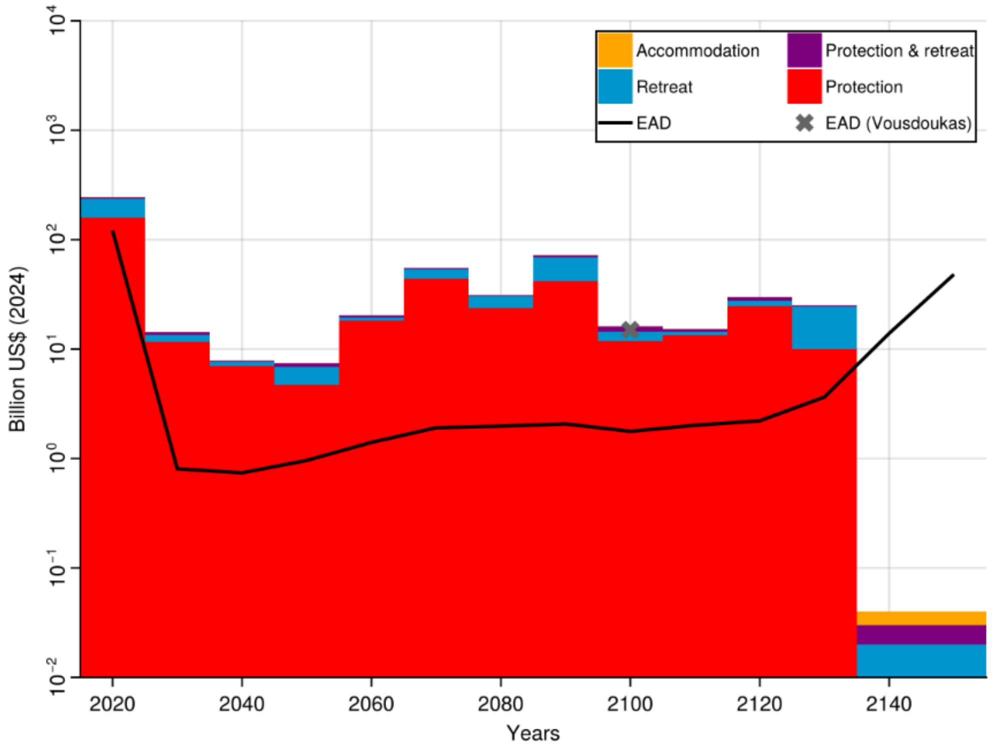

We also add to section 1.5 that: "Notably, half of the adaptation investments over the entire time horizon are concentrated in the first time step (Figure 2, note that this concentration is less apparent due to the logarithmic y-axis scale)."

- P6, L223: 'However, EAD rises again towards the end of the time horizon, as adaptation investments to maintain protection levels under rising sea levels cease after 2130'. I do not understand what you are trying to say here.

We thank the reviewer for pointing this out. We have clarified this sentence, and together with the updated Figure 2, the increase in EAD from 2130 onwards due to the absence of protection upgrades is now more apparent:

“Towards the end of the time horizon, EAD rises again because adaptation investments to maintain protection levels cease after 2130; with only two decades remaining until 2150, further protection upgrades are no longer cost-effective, leading to increasing residual flood damages under rising sea levels.”

- P5, L236: There is a typo in the word ‘extent’

Thank you for spotting this typo, it is corrected now.

Marjolijn Haasnoot,
Valeria Di Fant
Delft, 2025

References

Skerker, J. B., Zaniolo, M., Willebrand, K., Lickley, M., & Fletcher, S. M. (2023). Quantifying the value of learning for flexible water infrastructure planning. Water Resources Research, 59(6), e2022WR034412.

Truong, C., Malavasi, M., Li, H., Trück, S., & Shevchenko, P. V. (2024). Optimal dynamic climate adaptation pathways: a case study of New York City. Annals of Operations Research, 1-23.

Völz, V., Hinkel, J., Kupfer, S., MacPherson, L. R., & Wulff Norrby, C. J. (2024). Learning about sea level rise uncertainty improves coastal adaptation decisions. Earth's Future, 12(10), e2024EF004704.

Ward, P. J., Jongman, B., Aerts, J. C., Bates, P. D., Botzen, W. J., Diaz Loaiza, A., ... & Winsemius, H. C. (2017). A global framework for future costs and benefits of river-flood protection in urban areas. Nature climate change, 7(9), 642-646.

Reviewer #2 (Remarks to the Author):

I co-reviewed this manuscript with one of the reviewers who provided the listed reports. This is part of the Nature Communications initiative to facilitate training in peer review and to provide appropriate recognition for Early Career Researchers who co-review manuscripts.

Reviewer #3 (Remarks to the Author):

Thank you for the opportunity to review this manuscript, “The economically optimal mix and timing of coastal adaptation in Europe to 2150". The study advances research on coastal adaptation by providing large-scale quantitative evidence on the most economically optimal coastal adaptation pathways for coastal Europe. Unlike previous European coastal flood risk assessments, this work considers multiple adaptation strategies (i.e., protection, retreat, and accommodation), which enables the authors to identify points where the economically optimal strategy shifts (e.g., from accommodation to retreat). Notably, because the study includes multiple strategies and allows strategies to shift over time, the study projects a lower estimation of expected annual flood damages than previous efforts. The study focuses on a single hazard (coastal flooding) but does so at a higher spatial resolution than past efforts and considers 41,327 coastal floodplains.

Overall, this work advances the methods for adaptation decision-making science, and we recommend for publication with a few revisions to clarify interpretation of the results. We (co-reviewers) share a few opportunities for the authors to improve this manuscript below. The first group are edits we feel are important for the paper to be published, primarily focused on clarifying methods and a few revisions to figure captions. The second group are suggestions that we believe would strengthen the manuscript.

We thank reviewer #3 and #4 for this detailed review and are pleased to hear that they think this manuscript is advancing adaptation decision-making science. We address each question and comment of reviewer #3 and #4 below. We would like to thank reviewer #3 and reviewer #4 for helping us to improve our manuscript with their comments.

Concerns Needing Review

We had several questions about the methods and how choices about costs and limits affect interpretation of the results.

1. Authors describe accommodation as a binary variable indicating whether all homes in a 1-meter floodplain have been floodproofed to 1 meter in height. This appears to mean that accommodation, unlike retreat and protection, does not have a height variable higher than 1 meter. If this is incorrect, we ask the authors to clarify. If correct, the decision to limit accommodation to 1m high could be driving the study's findings that many areas in Europe will eventually shift from accommodation to retreat or protection (i.e., if accommodation were possible to a higher height, would this tipping point still occur?). We are not recommending additional analyses but that the authors discuss how this accommodation height affects the results and why 1m was selected as the limit. In the US, there are many structures that have undergone accommodation beyond 1 meter, so accommodation beyond this level is possible from engineering and economic perspectives.

We thank the reviewers for the important note that alternative definitions of accommodation could lead to different model outcomes. The reviewers are correct that we define accommodation as a binary variable indicating whether all homes in the 1-meter floodplain are floodproofed to a height of 1 meter. We chose this limit because the cost review by Aerts et al. (2018), which underpins our assumptions on accommodation costs, only reports dry floodproofing up to 1 meter in Europe (whereas cases in the United States have documented floodproofing up to 2 meters in Aerts et al., 2018). We have clarified this in the methods section as follows:

"We limit accommodation to 1 meter, as reported European cases do not exceed this level (Aerts et al., 2018)."

We also agree with the reviewers that allowing accommodation at higher elevations would affect model outcomes, particularly by delaying the tipping points at which accommodation is replaced by protection or retreat. To acknowledge this limitation, we added the following to the discussion:

"The range of adaptation options considered is limited; for example, accommodation for higher sea levels or advance (land claim or raising) are not included, which could delay adaptation tipping points or enable new pathways."

2. The authors estimate retreat costs at three times the local GDP per capita per migrant. We find that estimation to be low, as it is less than the median home value in many European countries. Retreat costs vary depending on the type of retreat (e.g., autonomous, government acquisitions, community relocations) but many government-invested retreat programs compensate

by home value, so a cost below median home value seems low. The authors note that they conducted a sensitivity analysis using retreat costs up to 6x GDP per capita. How did that sensitivity analysis affect the results? At 6x GDP, is an early investment in retreat still considered economically optimal?

We thank the reviewers for their valuable comments on retreat costs in relation to median home values. After reassessing our assumptions, we agree with the reviewers that 3× GDP per capita may be a relatively low medium estimate. We have therefore revised our retreat cost assumptions to 2× (low), 6× (medium), and 10× (high) GDP per capita, spanning the full range of observations reported by Lincke and Hinkel (2021), and have rerun all simulations accordingly. The default scenario SSP2-4.5 now uses increased retreat cost of 6x GDP per capita, resulting in retreat being applied on 23% of the European coastline and protection on 9% in 2150 (Table 1), compared to previously 26% and 6%, respectively. The early investment in retreat is not sensitive to the increase in retreat costs: in 99% of the coastline where retreat is implemented, it is still chosen in the very first time step.

3. One significant result that currently feels buried is that it is economically optimal to retreat from 99% of the retreated coast (26% total) in the first time step. (Although, as per our previous point, is this still true if retreat costs double?) How did the authors consider the short-term economic benefits gained from coastal development? That is, what are the lost economic opportunities associated with retreat at an early phase? Whether those losses are none or are outweighed by the gains in avoided damage from flooding, either way the result is surprising and merits highlighting. If the missed opportunity costs were not considered, that is a limitation worth noting in the discussion or methods.

We thank the reviewers for raising short-term opportunity costs of coastal development potentially challenging an early retreat from the coast. In our model, even if retreat costs are increased to 10xGDP per capita (instead of 6x), the percentage of floodplains where retreat is implemented in the first time step remains at 99%, and only a slight reduction in the total coastline applying retreat occurs (23.3% to 22.8%). We have added the following note in a new paragraph on limitations:

“In our model, retreat is implemented in the first time step for 99% of floodplains. While this approach may prevent new coastal developments that could lock floodplains into a protection pathway, potential coastal development opportunities, such as ports, are not represented in the model.”

4. A few minor suggestions for figure captions:

- There is some confusing text in the caption of Figure 5, including "shown in blue (green)".

We thank the reviewers for pointing out that the caption of Figure 5 was unclear, particularly the phrasing "shown in blue (green)." We have revised the caption to remove ambiguity and now spell out both sentences explicitly:

"Lower input parameters, shown in blue, correspond to SSP1-2.6, a discount rate of 1%, low adaptation costs for protection and retreat, and a reduction of initial protection heights by 20%. Higher input parameters, shown in green, correspond to SSP5-8.5, a discount rate of 5%, high adaptation costs for protection and retreat, and an increase of initial protection heights by 20%."

- Figure 1: It would be useful to describe in the caption that "height of retreat" means the height above sea level below which infrastructure is removed. This is stated in the methods section but is needed here for a reader to understand the figure.

We thank the reviewer for pointing out that the meaning of "height of retreat" was not clear in the caption of Figure 1. We have revised the caption to explicitly define this term so that the figure is self-contained and understandable without referring to the Methods section:

"The panels on the right show the corresponding economically optimal adaptation pathway, in terms of the heights of protection, retreat and accommodation options over time. Here, the height of retreat refers to the height above sea level below which assets and people are removed."

- Figure 2: the caption says "both" but there are three graphs, not two. Is the EAD line truly at $0 between 2030 and 2100? (From the text, it appears not, but the graph makes it look like $0.) If so, that is remarkable and a result worth highlighting. If not, it would be worth clarifying the actual scale of the EAD and that the line appears to reach 0 due to scaling on the graph. The caption also needs to explain the "X EAD Vousdoukas" to enable a reader to interpret the graph alone.

We thank the reviewers for pointing this out. We have updated the figure caption and rescaled the y-axis to a logarithmic scale, which now clearly shows that EAD does not reach zero between 2030 and 2100. The caption also now clarifies the meaning of the "X EAD (Vousdoukas)" marker, enabling readers to interpret the graph independently:

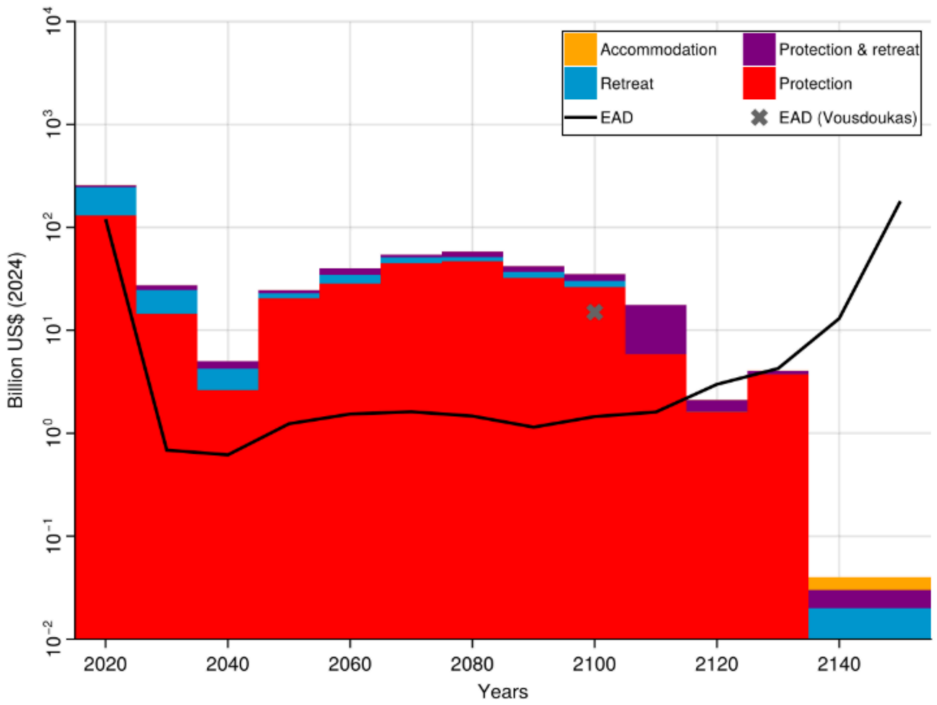

"Graphs showing adaptation investments for each adaptation option (stacked bar plots) and expected annual flood damage (EAD) (black line), shown undiscounted, over time and aggregated across all floodplains on a logarithmic scale, under SSP2-4.5. EAD (Vousdoukas) indicates the European EAD reported in Vousdoukas et al. [30]."

<u>Minor Notes / Suggestions</u>

1. We recommend revising the abstract to focus less on adaptation tipping points (ATP) and more on other surprising results. Although including ATP in the analysis is a methodological advance, the paper has fewer surprising findings about ATP. Reframing the abstract and introduction to highlight the most surprising and important results of the paper will help this work be read by the wider audience it deserves. Findings we found particularly interesting include:

We thank the reviewers for noting that some of our most surprising results were not highlighted in the abstract. While other reviewers acknowledged the focus on adaptation tipping points (ATP) and even suggested including additional ATP findings in the abstract, we have now revised it to balance results of the timing of actions, the percentage of coastline where different adaptation options are implemented, and (now fewer) findings on ATP. This revision aims to highlight a broader range of the study's results. We note that the 150-word abstract limit constrained our ability to include all suggested points. The revised abstract reads:

"Coastal adaptation to sea level rise involves combining and sequencing adaptation options into adaptation pathways that include adaptation tipping points - moments requiring a switch between options, often due to economic limits. While local case studies show costs depend on the economically optimal timing of actions and adaptation tipping points, this remains unexplored on global and continental scales. We present economically optimal adaptation pathways for Europe's coastal floodplains through 2150. For 95% of floodplains requiring adaptation, the optimal timing for initial actions is immediate due to current economic under-adaptation, a condition in which the costs of additional adaptation are outweighed by the reduction in expected flood damages. By 2150, retreat is economically optimal for 23% of the coastline—incurring lower total costs than protection, which is optimal for 9%. Adaptation tipping points, primarily driven by sea level rise, predominantly occur along the Mediterranean coastline, necessitating a switch from accommodation to either protection or retreat. Their timing depends on climate change, occurring 39 years earlier under higher climate change scenarios compared to lower ones."

The results show that retreat is economically optimal for one-fourth of Europe's coastline and that the adaptation costs for protection exceed those for retreat. This is a surprising finding and useful information for adaptation decision-makers, given Europe's history of choosing protection over other options.

a. The analysis shows that for 99% of coastlines needing adaptation, the optimal timing for initial actions is immediate (in the first time step), and the initial investments are significant (Figure 2). The idea that widespread action is needed urgently is not surprising, but the extreme nature of the results in this study are surprising and worth highlighting.
    i. This also raises a question: Did the authors place any limits on adaptation investments (e.g., a limit that nations do not invest more than 20% of their GDP in a given decade)? Or a sensitivity analysis to see what happens if nations do not invest in adaptation in the first time step? Additional analyses are not required, but we were surprised by the extreme costs in the first step (Figure 2) and wondered if that level of adaptation investment is

economically or politically possible. If not, the analysis provides an interesting hypothetical but not an applicable guide.

We did not impose explicit limits on adaptation investments, apart from constraining the maximum adaptation height to the 1-in-10,000-year extreme water level plus projected sea-level rise in 2150, and the overall cost-minimization requirement of the objective function. We acknowledge that the adaptation investments in the first time step (2020) appear relatively large. Specifically, the model suggests investments of US$ 242 billion in 2020, which corresponds to 1.1% of Europe's GDP in 2023. We thank the reviewers for highlighting this point, as it is important to put these numbers in context. We have therefore added a reference to these values in the cost section:

"Over the first decade (2020–2030), EAD accumulates to US$ 1,210 billion (5.4% of Europe's GDP in 2023), far exceeding the one-time adaptation investments of US$ 242 billion in 2020 (1.1% of Europe's GDP in 2023)."

b. Table 1: The final column appears to compare the costs of adaptation and flood damages (totaled over 130 years) to national GDP in 2023. This temporal mismatch makes it unclear how to use that final column. What does it tell us that adaptation costs and flood damages combined over the coming century will cost 8.2% of Europe's GDP? Would it not be more relevant to compare the adaptation costs in the first time step to 2023 GDP? Or to compare the coming century of adaptation and flood damages to the coming century of GDP? Given the extreme adaptation costs that are optimal in the first time step, it would be fascinating to see a comparison of the recommended adaptation investments in the first time step for each country relative to that nation's GDP. That would provide a very useful insight into the feasibility of the recommended investments.

We thank the reviewers for this insightful suggestion. In response, we have updated Table 1 to report the absolute adaptation costs in the first time step (2020) along with their percentage relative to each country's GDP in 2023. This provides a more immediate and practical perspective on the feasibility of the recommended investments. We have also incorporated this perspective when addressing the previous comment regarding high initial adaptation costs.

c. The phasing of retreat to protection was surprising. It makes sense, especially where retreat to higher ground results in denser infrastructure that then merits protection. However, this phasing is rarely discussed in the adaptation literature and is a notable contribution to discussions around adaptation pathways.
    i. In Lines 292-299, the authors discuss retreat as a “last resort” when no ATP are reached after retreat. While we appreciate the author’s point about protection at higher elevations after (or in coordination with) retreat, we caution on the "last resort" language because of the spatial issue. Namely, protection at higher elevation after retreat has occurred at lower elevation still involved retreat as a “last resort” for the people living at those lower elevations. A controversial example from the United States is the relocation of a tribal community on Isle de Jean Charles. A system of protected levees was built inland of the island (and at higher elevation), leaving the islanders with few options other than retreat. The fact that the region combined protection, accommodation, and retreat does not make retreat less of a last resort for this Tribe. We recommend a minor edit to change the last resort language to phrasing that acknowledges the combination of strategies.

We thank the reviewers for highlighting this important perspective. We agree that the term “last resort” requires careful framing, as retreat may still be the only option for people at lower elevations even when protection is applied at higher elevations. We have revised the manuscript to clarify that “last resort” is a direct quotation and to emphasize that our model applies a combination of strategies:

“In contrast to the generic adaptation pathways, in which retreat occurs as the “last resort” implemented over time (Haasnoot et al., 2019), our results show that retreat is sometimes followed by, and combined with, protection.”

2. The authors say that 68% of the total length of Europe's coastline will not require adaptation because it is not exposed to flood risks and SLR. Does the analysis also consider coastal erosion rates (we see a note about vertical land movement in section 3.2.2 but no discussion of erosion)? Or interactions with inland and riverine flooding? (In London and much of the Netherlands, for example, the interaction between coastal and riverine flooding is a major consideration in adaptation planning.) Our suggestion is not to include erosion or riverine flooding but to provide some discussion on how the authors think inclusion of additional hazards might change the results of their analysis.

We thank the reviewers for raising this important point. We have incorporated the following discussion into the manuscript:

“We focus solely on coastal flooding, whereas other hazards, such as coastal erosion, heavy precipitation, and riverine flooding, are not considered. These hazards could require additional adaptation investments; for instance, global dike investments for riverine flooding could increase our adaptation costs in the first time step by roughly 36% (Ward et al, 2017). Other hazards may also interact with the adaptation options considered here - for example, coastal protection could exacerbate erosion or complicate drainage during heavy precipitation.”

3. Why did the authors choose London, Venice, and Thessaloniki as examples? Venice and London, in particular, are known for being unusual in terms of their exposure (e.g., London being affected by coastal and riverine flooding and being a somewhat non-traditional “coastal” city; and Venice being a heritage icon). This may lead readers (as it did to us) to fixate on the particulars of those cities, whereas a less iconic city might help illustrate the authors’ analysis and the value of the findings more clearly.

We thank the reviewers for this comment. We now begin our Results section with a brief introduction to the three city examples, explaining why they were selected and highlighting their differences. These cities were chosen to illustrate a range of geographic and socio-economic characteristics, while also being familiar to a broad readership, including readers outside Europe. This framing helps clarify that the choice of London, Venice, and Thessaloniki is intended to demonstrate contrasting adaptation challenges rather than focus on their unique or iconic status:

“To illustrate the general nature of our approach, we first present results for three major European cities under SSP2-4.5, representing different geographic and socio-economic characteristics in terms of extreme sea levels, population density, and current protection levels. The cities are: London, a mega-city with very high coastal flood protection; Venice, a large city with medium protection levels and UNESCO World Heritage status; and Thessaloniki, a large city without large coastal flood protection today. In our model, each city and its surrounding regions include multiple floodplains, spanning densely populated urban areas as well as rural zones with low asset values. We focus on two representative floodplains per city (Figure1).”

4. In the introduction, the authors note that ATP is driven by economic and social factors. Line 200-202 notes that ATP “can be completely attributed to SLR” (within the model) because it does not occur in a counterfactual situation. Both of these

statements may be true, but it would be interesting to see the authors discuss why they think SLR drives ATP: why projected population and GDP growth rates, for example, were insufficient to drive ATP.

We thank the reviewers for this insightful comment. We confirm that in a counterfactual scenario including socio-economic development (SED)—i.e., projected population and GDP growth—but excluding sea-level rise (SLR), no ATP occurred in our model (Figure S1). We now explicitly highlight the effect of SED on ATP in the revised Results section:

"Comparing a counterfactual scenario without socio-economic development (SED) to SSP2-4.5 (Figure S1) further illustrates that SED influences the type of ATP. Without SED, only very few switches from accommodation to protection occur because lower asset values and reduced EAD do not justify protection along the same coastal stretches. In the absence of SLR, SED alone is insufficient to trigger any ATP in our model. Although asset growth under SED increases EAD, it does not raise the benefit–cost ratios of protection above those of accommodation; this is achieved only through SLR, which directly increases the hazard component and therefore significantly raises EAD."

We hope that this review is useful to the authors, and we look forward to the publication of this manuscript.

Reviewer #4 (Remarks to the Author):

I co-reviewed this manuscript with one of the reviewers who provided the listed reports. This is part of the Nature Communications initiative to facilitate training in peer review and to provide appropriate recognition for Early Career Researchers who co-review manuscripts.

References

Ward, P. J., Jongman, B., Aerts, J. C., Bates, P. D., Botzen, W. J., Diaz Loaiza, A., ... & Winsemius, H. C. (2017). A global framework for future costs and benefits of river-flood protection in urban areas. Nature climate change, 7(9), 642-646.
